# A retrospective survey on injuries in Croatian football/soccer referees

**DOI:** 10.1186/1471-2474-14-88

**Published:** 2013-03-11

**Authors:** Goran Gabrilo, Marko Ostojic, Kemal Idrizovic, Bozidar Novosel, Damir Sekulic

**Affiliations:** 1 Faculty of Kinesiology, University of Split, Teslina 6, Split 21000, Croatia; 2 University Clinical Hospital Mostar, Kralja Tvrtka bb, Mostar 63000, Bosnia and Herzegovina; 3 Faculty of Sport and Physical Education, University of Monte Negro, Podgorica, Monte Negro; 4 General Hospital Varazdin, Mestrovica bb, Varazdin 42000, Croatia; 5 NIHON doo, Spinutska 65, Split 21000, Croatia

**Keywords:** Injury status, Quality, Match, Fitness test

## Abstract

**Background:**

Injury among soccer referees is rarely studied, especially with regard to differences in the quality level of the refereeing. Additionally, we have found no study that has reported injury occurrence during official physical fitness testing for soccer referees. The aim of this study was to investigate the frequency, type and consequences of match-related and fitness-testing related injuries among soccer referees of different competitive levels.

**Methods:**

We studied 342 soccer referees (all males; mean age 32.9 ± 5.02 years). The study was retrospective, and a self-administered questionnaire was used. In the first phase of the study, the questionnaire was tested for its reliability and applicability. The questionnaire included morphological/anthropometric data, refereeing variables, and musculoskeletal disorders together with the consequences.

**Results:**

The sample comprised 157 main referees (MR; mean age 31.4 ± 4.9 years) and 185 assistant referees (AR; mean age 34.1 ± 5.1 years) divided into: international level (Union of European Football Associations-UEFA) referees (N = 18; 6 MRs; 12 ARs) ; 1^st^ (N = 78; 31 MRs; 47 ARs), 2^nd^ (N = 91; 45 MRs; 46 ARs); or 3^rd^ national level referees (N = 155; 75 MRs; 80 ARs). In total, 29% (95%CI: 0.23–0.37) of the MRs and 30% (95%CI: 0.22–0.36) of the ARs had experienced an injury during the previous year, while 13% (95%CI: 0.05–0.14) of the MRs, and 19% (95%CI: 0.14–0.25) of the ARs suffered from an injury that occurred during fitness testing. There was an obvious increase in injury severity as the refereeing advanced at the national level, but the UEFA referees were the least injured of all referees. The results showed a relatively high prevalence of injuries to the upper leg (i.e., quadriceps and hamstrings) during physical fitness testing for all but the UEFA referees. During game refereeing, the ankles and lower legs were the most commonly injured regions. The MRs primarily injured their ankles. The ARs experienced lower leg and lower back disorders. However, the overall injury rate was equal for both groups, with 5.29 (95%CI: 2.23–8.30) and 4.58 (95%CI: 2.63–6.54) injuries per 1000 hours of refereeing for MRs and ARs, respectively.

**Conclusion:**

In addition to the reported risk of injury during soccer games, physical fitness testing should be classified as a risk for injury among soccer referees. Special attention should be given to (I) lower leg injuries during games and (II) upper leg injuries during physical fitness tests. A higher physical fitness level and a qualitative approach to training are recognized as protective factors against injury. Subsequent studies should investigate the specific predictors of injuries among referees.

## Background

With more than 240.000.000 registered players (17% female), soccer (i.e., European “football”) is the most popular sport in the world today [1-4]. Although the players and their mastery of the sport are the primary reasons for soccer’s popularity [5,6], the soccer referees are an inseparable factor of this sport [7-10]. The Fédération Internationale de Football Association (FIFA) reported more than 800.000 registered soccer referees and assistant referees [11]). The referees’ activities during the game are regularly studied and reported [12-15]. In addition, their fitness level is a highly important factor, and it is officially tested [16-20]. An appropriate fitness status is a mandatory prerequisite for any referee at advanced competitive levels [17,21,22]. The overall physical demands on the referees are considered similar to those on the soccer players. In short, a referee covers a distance of 9 to 13 km, with 4%–18% of physical activity consisting of high-intensity running; elite referees may perform up to 1270 changes in activity during a game and make more than 130 decisions [14,15,23]. However, high-level referees are 15–20 years older than the players and, in most cases, are not professionals and cannot be substituted during the game. Evidently, an appropriate health status is one of the most important aspects of successful refereeing. The high demands of the game and the stress it places on the musculoskeletal and cardio-pulmonary systems increase the risk of injury. One of the first studies that investigated this problem was that of Fauno and his associates [24], where the authors recognized the problem and investigated the effect of shock-absorbing heel inserts on the incidence of soreness. Bizzini et al. [25] studied referees selected for the 2006 FIFA World Cup and reported the types of injuries, as well as the location, characteristics, type, frequency, and consequences of the complaints and the type of treatment offered. More than 40% of the referees reported an injury, more than 60% had musculoskeletal complaints during their career, and there was a mean rate of 20.8 injuries per 1000 match hours. Almost 50% of all female soccer referees participating in the Women’s World Cup 2007 reported that they had been injured during their career, with a rate of 34.7 match injuries per 1000 match hours during the World Cup [26]. However, those data are only partially generalizable to referees from lower levels. Studies performed on Swiss referees reported a rate of 22.5% to 44% of injured referees [27,28]. In a recent review Weston et al. concluded that high training loads combined with increasing age could, in part, explain the incidence of non-contact match injuries (18 injuries per 1000 match hours), with lower leg muscle strains being the most common type of non-contact injuries in referees [29].

In one of the rare prospective studies on this topic, performed on Irish referees [30], the authors found 8.8 injuries/1000 hours of training and 16.4 injuries/1000 hours for match officiating. However, the risk of injury varies considerably based on the standards of the game of which a referee has been placed in charge. Additionally, previous studies did not report data on injuries that happened as a result of official physical fitness testing of soccer referees.

The aim of this study was to describe the frequency, type and consequences of injuries among Croatian soccer referees. Specifically, we studied the problems regarding (A) the various levels of refereeing quality, (B) the differences between the main and assistant referees, and (C) the injuries that occurred during games and those that occurred during physical fitness testing.

## Methods

### Design and subjects

Prior to this retrospective study, we contacted the Croatian Football Federation and obtained formal permission for the study. A self-administered questionnaire that had been previously used in similar studies and obtained from the authors of those studies [25-28] was used as a measurement tool (Additional file 1). Based on previous studies [25-28], the content validity of the questionnaire is therefore presumed. Additionally, following translation into Croatian, the questionnaire was checked for its clarity and content validity by two MDs (both sports-medicine specialists) and one high-level soccer referee (first author of the paper). The first phase of the experiment consisted of an evaluation of the reliability of the translated questionnaire. Twenty-two referees were asked to complete the questionnaire twice (a test-retest procedure, with a period of 10 days between the test and re-test). The reliability was checked throughout the test-retest correlation (for ordinal variables) and the analysis of equally answered questions (for ordinal and nominal variables) [31]. The reliability test showed the high reliability of the questionnaire translated into Croatian. Briefly, the test-retest correlation for ordinal variables ranged from 0.80 (for injury consequences for the whole career) to 0.99 (refereeing experience). The consistency of the test-retest answers was 90 to 100%, and differences were only found for some of the questions. Because the most common and the greatest disagreements between the test and retest results were found for injuries and consequences throughout the whole refereeing career, in this investigation, we have focused on the previous year, the most recent refereeing match and physical fitness testing. The discriminative validity of the questionnaire was estimated throughout this investigation by discriminating MRs and ARS and differentiating referees of different levels [32]. The survey was administered at seven specialized soccer referee seminars from April to July of 2011. Using the multimedia presentation (see Additional file 2) the first author informed subjects about the purpose of the study, explained them the questionnaire design, and other important issues (definition of injuries; anonymity, testing protocol, etc.). The participants consisted of 342 soccer referees (all males, engaged in outdoor soccer; mean age 32.9), comprising more than 90% of all licensed Croatian soccer referees. Practically all subjects who were present at the seminars from April to July of 2011 (90% of all Croatian national level referees) were asked to participate in the study, and the response rate was more than 95%. Only a few examinees did not answer all questions. The study objective was to assess a representative sample of Croatian soccer referees of various competitive levels. Subjects completed the questionnaire in groups of 15 to 20 respondents, and the examiner (the first author) was available at all times for questions and answers. The participants were informed that they could refuse participation and withdraw from the study at any time, for any reason, and their informed consent was obtained. Participation was anonymous (except for those examinees included in the test-retest procedure for reliability testing), and no personal data regarding date of birth, city of residence and/or any other data that could be directly connected to an individual were included in the questionnaire. The answer options were presented as multiple-choice, closed-ended responses for most of the questions. Prior to the study, ethical approval was obtained from the Ethical Board of the Faculty of Kinesiology, University of Split, Croatia.

### Variables

The questionnaire consisted of the following sections: (1) morphological-anthropometric data; (2) refereeing variables; and (3) musculoskeletal injuries together with the consequences of injury.

Morphological anthropometric data: Subjects reported body height (BH) and body mass (BM) according to the official measurements performed during physical fitness testing (a day or two before the questioning was performed), and we calculated the body mass index (BMI).

Refereeing variables: The subjects were grouped into main and assistant referee groups. We asked the subjects about their experience in refereeing (e.g., how long ago did they receive their official license), the highest current level of refereeing achieved (four point scale: UEFA level, 1^st^ national, 2^nd^ national, 3^rd^ national level), the number of games refereed during the last year, and the average training hours per week (during the preseason and during the season, two separate questions).

Musculoskeletal injuries and consequences: The respondents were asked about the occurrence of injuries and musculoskeletal complaints for the most recent match, the last year, and the entire career, as originally suggested by the authors [25-28]. However, in this study we have reported only data on injuries for the most recent match and the last year. In addition to the items on the original questionnaire, the subjects were asked if they had any injuries or complaints during the physical testing. If the answer was yes, the subject was asked for the location of the injury (22 choices, such as head, neck, etc.), the type of injury (15 choices, such as dislocation, contusion, tendinitis, etc.), the length of recovery (how many days the consequences of the injury were felt), and absence from training (days) and refereeing (days). In the case of multiple injuries, all injuries were reported separately.

An injury was defined as “any physical complaint sustained as a result of refereeing and training, irrespective of the need for medical attention or time lost from activity (refereeing or training)”, which was adopted from Fuller et al. [33]. To calculate the ratio of injuries per 1000 hours of match during the last year (see enclosed questionnaire; page 5, section 4; 2^nd^ question), the hours were determined as the total number of games refereed throughout the last year x 1.5 hours (90 minutes of official game with no extra time). The ratio of injuries per 1000 hrs of last year refereeing is calculated separately for MRs and ARs according to their quality level.

### Statistics

For parametric variables (age, experience, etc.), the means and standard deviations were reported. For nonparametric measures (i.e., nominal and ordinal variables), the frequencies and proportions, together with 95% confidence intervals in some cases [34], were calculated. Depending on the parametric/nonparametric nature of the variables, we applied a univariate analysis of variance (ANOVA) with the Schefee post-hoc analysis and/or the Kruskal-Wallis ANOVA [35] to establish the differences between (a) the main and assistant referees and (b) the referees of different levels (separately for main and assistant referees). The responses from the open-ended questions and other comments by the subjects were transcribed and further analyzed for common themes. A value of P < 0.05 was considered statistically significant. The statistical analyses were performed using Statsoft’s Statistica version 10.

## Results

Of 342 subjects, the 157 were main referees (MRs; mean age 31.5 years), and 185 were assistant referees (ARs; mean age 34.1 years). The ARs were significantly (P < 0.05) older than the MRs, but only when considering those involved in the 1^st^ and 3^rd^ national leagues. The 1^st^ and 2^nd^ national league MRs were significantly (P < 0.05) taller than the ARs. The ARs from the 2^nd^ and 3^rd^ league had significantly (P < 0.05) longer experience than their MR peers. The UEFA referees were the most experienced in refereeing, followed by the 1^st^ league MRs. The MRs were significantly (P < 0.05) taller than the ARs. Among the ARs, the UEFA referees were the oldest. The 3^rd^ league ARs had the highest BMI of all the groups (Table 1).

**Table 1:** Descriptive statistics for general data of examinees (M-mean, SD-standard deviation), and ANOVA differences between match referees (MR) and assistant referees (AR) within the same competitive level (* denotes significant differences), and within MR and AR between the different competitive levels (^**¥ **^**denotes significant differences)**

		**Total (n = 342)**		**UEFA (n = 18)**		**1**^**st **^**(n = 78)**		**2**^**nd **^**(n = 91)**		**3**^**rd **^**(n = 155)**	
	**MR (n = 157)**	**AR (n = 185)**	**MR (n = 6)**	**AR (n = 12)**	**MR (n = 31)**	**AR (n = 47)**	**MR (n = 45)**	**AR (n = 46)**	**MR (n = 75)**	**AR (n = 80)**	
	**M ± SD**	**M ± SD**	**M ± SD**	**M ± SD**	**M ± SD**	**M ± SD**	**M ± SD**	**M ± SD**	**M ± SD**	**M ± SD**	
Age (years)	31.4 ± 4.88	34.11 ± 5.08	34.67 ± 2.5	38.25 ± 4.45	33.65 ± 4.46	35.8 ± 4.15*	30.59 ± 4.42	31.98 ± 4.97	30.88 ± 5.16^¥^	33.71 ± 5.11*^¥^
Experience (years)	10.79 ± 3.26	12.52 ± 3.43*	17 ± 2.28	17 ± 2.98	13.24 ± 2.57	14.33 ± 2.62	9.47 ± 2.87	11.31 ± 3.82*	10.15 ± 2.71^¥^	11.4 ± 2.54*^¥^
BH (cm)	183.4 ± 5.75	181.41 ± 6.23*	181 ± 3.52	183.17 ± 8.11	185.69 ± 6.07	182.78 ± 6.28*	183.6 ± 5.08	180.4 ± 5.69*	182.59 ± 5.96	180.91 ± 6.158
BM (kg)	83.44 ± 7.24	82.04 ± 7.45	81.5 ± 2.35	81.5 ± 7.45	85.93 ± 7.46	83.89 ± 7.09	82.06 ± 7.35	79.49 ± 7.04	83.47 ± 7.19	82.5 ± 7.62^¥^
BMI (kg/m2)	24.77 ± 1.35	24.9 ± 1.44	24.88 ± 0.42	24.28 ± 1.37	24.88 ± 1.23	25.07 ± 0.95	24.31 ± 1.45	24.41 ± 1.71	25 ± 1.32	25.17 ± 1.46^¥^

LEGEND: Age-age of the subjects, Experience-time involved in the soccer refereeing, BH-body height, BM-body mass, BMI-body mass index.

In general, 60 (17%), 101 (29%) and 56 (16%) referees reported injuries during the last match, during the last 12 months and during physical fitness testing, respectively, corresponding to 0.19, 0.41 and 0.22 injuries, respectively, per subject. Excluding the UEFA MRs, who were the least commonly injured, the higher level referees were more frequently injured than their peers who participate in matches at the lower levels. When comparing the ARs for injuries between levels, the 1^st^ league referees were the most commonly injured during fitness testing, with a significant difference (P < 0.05) between the 1^st^ and 3^rd^ leagues. There was an overall significant (P < 0.05) Kruskal Wallis difference in the 12-month injury status between the different levels of ARs, with an observable trend toward fewer injuries in national lower level refereeing (Table 2).

**Table 2:** Prevalence of injuries (frequency-f; percentage-%; 95% CI-95% confidence interval) among soccer referees during the last match, the last year and during fitness testing, and Kruskal Wallis analysis of the differences (* denotes significant differences) between qualitative levels (UEFA referees-UEFA, 1^**st **^**National league-1**^**st**^**, 2**^**nd **^**National league-2**^**nd**^**, 3**^**rd **^**National league), within match referees (MR), and within assistant referees (AR)**

	**Last match**		**Last year**		**During fitness testing**	
	**f (%)**	**95% CI**	**f (%)**	**95% CI**	**f (%)**	**95% CI**
MR	29 (18)	0.13–0.25	46(29)*	0.23–0.37	21(13)	0.05–0.14
AR	31 (17)	0.12–0.22	55(30)*	0.23–0.37	35(19)*	0.14–0.25
UEFA MR	0(0)	-	3(50)	0.19–0.81	0(0)	-
UEFA AR	0(0)	-	1(8)	0.01–0.35	3(25)	0.09–0.53
1^st^ MR	8(26)	0.13–0.43	12(39)	0.24–0.56	7(23)	0.11–0.40
1^st^ AR	11(23)	0.13–0.37	21(45)	0.31–0.58	17(36)	0.24–0.50
2^nd^ MR	8(17)	0.01–0.31	17(38)	0.25–0.52	8(18)	0.09–0.31
2^nd^ AR	8(17)	0.09–0.30	14(30)	0.19–0.45	9(20)	0.10–0.33
3^rd^ MR	13(17)	0.10–0.27	14(19)	0.11–0.29	6(8)	0.04–0.16
3^rd^ AR	12(15)	0.12–0.29	19(24)	0.16–0.34	6(8)	0.03–0.15

As a result of injuries that occurred during the last match, the 1^st^ league MRs abstained from training and refereeing for the shortest time. For the injuries that occurred during the last year, the UEFA referees abstained from training for the shortest time (18 days on average). The longest break because of injuries that occurred during physical fitness testing was noted for the 3^rd^ league (79 days), followed by the 1^st^ league (41 days) and 2^nd^ league referees (22 days). It must be stressed that UEFA MRs did not experience any injuries during physical fitness testing (Table 3).

**Table 3:** **Descriptive statistics for consequences of injury which occurred during the last match, last year and during fitness testing among match referees (n-number of subjects in each group, M-mean, SD-standard deviation) for different competitive levels (UEFA referees-UEFA, 1**^**st **^**National league-1**^**st**^**, 2**^**nd **^**National league-2**^**nd**^**, 3**^**rd **^**National league)**

	**Total (n = 342)**	**UEFA (n = 18)**	**1**^**st **^**(n = 78)**	**2**^**nd **^**(n = 91)**	**3**^**rd **^**(n = 155)**
	**M ± SD**	**M ± SD**	**M ± SD**	**M ± SD**	**M ± SD**
Last match (injured)	n = 29	n = 0	n = 8	n = 8	n = 13
Recovery (days)	36.5 ± 43.42		30.38 ± 29.14	52.5 ± 45.17	33.11 ± 55.91
No training (days)	36.38 ± 48.47		21.43 ± 18.49	39 ± 32.86	46.73 ± 66.62
No refereeing (days)	58.38 ± 64.11		42.33 ± 42.85	54.67 ± 57.14	74.67 ± 83.96
Last year (injured)	n = 46	n = 3	n = 12	n = 17	n = 14
Recovery (days)	53.55 ± 73.43	76 ± 90.16	73.4 ± 104.73	51.63 ± 68.5	35.46 ± 46.5
No training (days)	35.35 ± 35.31	17.5 ± 4.95	34.5 ± 22.48	38.46 ± 34.49	35.5 ± 44.96
No refereeing (days)	42.59 ± 48.67	20.5 ± 0.71	20.4 ± 8.76	36.11 ± 35.99	78.17 ± 74.38
Fitness testing (injured)	n = 21	n = 0	n = 7	n = 8	n = 6
Recovery (days)	39.94 ± 57.69		36.57 ± 28.56	59 ± 101.29	22 ± 16.37
No training (days)	32.25 ± 25.73		31.33 ± 22.37	37.67 ± 32.21	25.5 ± 25.04
No refereeing (days)	43.11 ± 47.13		41 ± 33.53	22.33 ± 13.28	78.5 ± 101.12

For the ARs, the recovery from injuries that occurred during the last match ranged from 30 (2^nd^ league) to 81 days (3^rd^ league) on average. As a result of injuries that happened during the last year, the shortest period with no training was presented by the ARs at the UEFA level. Injuries occurring during the last refereed game did not result in any pause from refereeing for the UEFA ARs. However, an average of 21, 37 and 87 days elapsed before the 3^rd^, 1^st^ and 2^nd^ league ARs resumed their duties, respectively. As a result of fitness testing injury, the 1^st^ league ARs abstained from their training for a relatively brief period of time (26 days) when compared with their 2^nd^ league colleagues, who abstained from their training for 40 days (Table 4).

**Table 4:** **Descriptive statistics for the consequences of injury which occurred during the last match, last year and during fitness testing among assistant referees (n-number of subjects in each group, M-mean, SD-standard deviation) for different competitive levels (UEFA referees-UEFA, 1**^**st **^**National league-1**^**st**^**, 2**^**nd **^**National league-2**^**nd**^**, 3**^**rd **^**National league)**

	**Total (n = 342)**	**UEFA (n = 18)**	**1**^**st **^**(n = 78)**	**2**^**nd **^**(n = 91)**	**3**^**rd **^**(n = 155)**
	**M ± SD**	**M ± SD**	**M ± SD**	**M ± SD**	**M ± SD**
Last match (injured)	n = 31	n = 0	n = 11	n = 8	n = 12
Recovery (days)	54.91 ± 57.96		43.43 ± 40.41	29.83 ± 30.31	80.56 ± 75.45
No training (days)	41.64 ± 53.23		33.67 ± 30.21	44.5 ± 57.23	44.75 ± 67.08
No refereeing (days)	78.33 ± 102.61		66.6 ± 64.67	104 ± 174.27	71 ± 84.76
Last year (injured)	n = 55	n = 1	n = 21	n = 14	n = 19
Recovery (days)	48.5 ± 87.19	6 ± 0	75 ± 119.55	37.75 ± 50.07	12.43 ± 9.9
No training (days)	36.85 ± 56.88	6 ± 0	49.15 ± 78.83	45.2 ± 50.5	15.6 ± 11.06
No refereeing (days)	45.87 ± 74.26		37.09 ± 29.84	87.33 ± 139.27	20.5 ± 9.01
Fitness testing (injured)	n = 35	n = 3	n = 17	n = 9	n = 6
Recovery (days)	44.86 ± 91.31	13.67 ± 7.51	69.6 ± 120.7	13.83 ± 12.86	22 ± 9.24
No training (days)	26.75 ± 34.13	10.33 ± 6.35	26 ± 20.21	40.14 ± 62.61	18.25 ± 7.85
No refereeing (days)	48.74 ± 49.95	24.5 ± 14.85	59.44 ± 50.15	55.4 ± 70.36	21.67 ± 8.02

The Achilles tendon, knee, ankle and lower leg were the most commonly injured body regions. The MRs injured the Achilles tendon, thigh, and ankle most commonly, while the ARs experienced more lower leg, lower back and shoulder injuries. There was no evident difference between the type of injuries that occurred in the last 12 months and those that occurred during the last match refereed. During fitness testing, thigh and groin injuries prevailed (Table 5).

**Table 5:** **Location of injuries (frequencies-f; percentage-%) among soccer referees on the last match, during the last year and during fitness testing, for match referees (MR), and assistant referees (AR); and for different competitive levels (UEFA referees-UEFA, 1**^**st **^**National league-1**^**st**^**, 2**^**nd **^**National league-2**^**nd**^**, 3**^**rd **^**National league**

	**Total n = 342**	**MR n = 157**	**AR n = 185**	**UEFA n = 18**	**1**^**st **^**n = 78**	**2**^**nd **^**n = 91**	**3**^**rd **^**n = 155**
	**f (%)**	**f (%)**	**f (%)**	**f (%)**	**f (%)**	**f (%)**	**f (%)**
Last match (n = 67 injuries)							
Elbow	1 (1)	0(0)	1(3)	0(0)	0(0)	0(0)	1(4)
Lower back	4 (6)	0(0)	4(11)	0(0)	1(4)	1(6)	2(8)
Abdominal, hip and groin area	10(14)	4(13)	6(17)	0(0)	1(4)	5(28)	4(16)
Thigh	5 (7)	2(7)	3(8)	0(0)	2(9)	2(11)	1(4)
Knee	10 (15)	4(14)	6(16)	0(0)	3(13)	3(17)	4(15)
Lower leg	9 (13)	2(7)	7(19)	0(0)	4(17)	0(0)	5(19)
Achilles tendon	17 (25)	8(28)	9(24)	0(0)	6(26)	6(33)	5(19)
Ankle and foot	11 (16)	9(31)	1(3)	0(0)	6(26)	1(6)	4(15)
Last 12 months (n = 120 injuries)							
Head	1 (1)	1(2)	0(0)	0(0)	0(0)	0(0)	1(3)
Shoulder	3 (3)	0(0)	3(5)	0(0)	1(3)	1(2)	1(3)
Lower back	14 (12)	4(7)	10(17)	0(0)	4(11)	7(16)	3(9)
Abdominal, hip and groin area	17 (14)	9(14)	8(13)	2(34)	0(0)	7(14)	5(15)
Thigh	10 (8)	8(13)	2(3)	1(17)	4(11)	3(7)	2(6)
Knee	15 (13)	8(13)	7(12)	0(0)	2(5)	9(20)	4(12)
Lower leg	21 (18)	5(8)	16(27)	2(33)	5(14)	5(11)	9(27)
Achilles tendon	22 (18)	15(25)	7(12)	1(17)	12(32)	6(14)	3(9)
Ankle and foot	17 (14)	11(18)	6(10)	0(0)	6(16)	6(13)	5(15)
During fitness testing (n = 57 injuries)							
Head	3 (5)	0(0)	3(9)	0(0)	2(8)	1(6)	0(0)
Lower back	4 (7)	2(10)	2(6)	0(0)	1(4)	2(12)	1(8)
Abdominal, hip and groin area	13 (23)	4(20)	9(26)	2(22)	4(17)	5(30)	2(16)
Thigh	15 (26)	8(38)	7(20)	1(11)	8(33)	3(18)	3(25)
Knee	4 (7)	1(5)	3(9)	0(0)	2(8)	1(6)	1(8)
Lower leg	8 (15)	2(10)	6(17)	0(0)	1(4)	4(24)	3(25)
Achilles tendon	8 (14)	3(14)	5(14)	0(0)	6(25)	1(6)	1(8)
Ankle	1 (2)	1(5)	0(0)	0(0)	0(0)	0(0)	1(8)

Muscle strain was more common during fitness testing than during a match, but other types of injury prevailed during a match (Table 6).

**Table 6:** **Type of injuries (frequencies-f; percentage-%) among soccer referees on the last match, during the last year and during fitness testing, for match referees (MR), and assistant referees (AR); and for different competitive levels (UEFA referees-UEFA, 1**^**st **^**National league-1**^**st**^**, 2**^**nd **^**National league-2**^**nd**^**, 3**^**rd **^**National league**

	**Total n = 342**	**MR n = 159**	**AR n = 183**	**UEFA n = 18**	**1**^**st **^**n = 78**	**2**^**nd **^**n = 91**	**3**^**rd **^**n = 155**
	**f (%)**	**f (%)**	**f (%)**	**f (%)**	**f (%)**	**f (%)**	**f (%)**
Last match (n = 65 injuries)							
Fracture	1 (2)	0(0)	1(3)	0(0)	0(0)	0(0)	1(4)
Dislocation	1 (2)	0(0)	1(0)	0(0)	0(0)	1(5)	0(0)
Sprain	8 (12)	7(22)	1(0)	0(0)	3(9)	1(5)	4(17)
Ligament injury	8 (12)	4(13)	4(0)	0(0)	1(3)	3(15)	4(17)
Lesion of meniscus or cartilage	5 (8)	2(6)	3(0)	0(0)	2(6)	1(5)	2(8)
Muscle rupture/tear	4 (6)	2(6)	2(0)	0(0)	2(6)	1(5)	1(4)
Muscle strain	11 (17)	3(9)	8(0)	0(0)	5(16)	2(10)	4(17)
Tendon injury/strain	16 (25)	10(31)	6(0)	0(0)	2(6)	8(40)	6(25)
Tendinitis/bursitis	8 (12)	4(13)	4(0)	0(0)	5(16)	1(5)	2(8)
Contusion/hematoma	2 (3)	0(0)	1(0)	0(0)	1(3)	1(5)	0(0)
Laceration	1 (2)	0(0)	1(0)	0(0)	0(0)	1(5)	0(0)
Last 12 months (n = 110 injuries)							
Concussion	1 (1)	0(0)	1(2)	0(0)	0(0)	0(0)	1(3)
Fracture	1 (1)	0(0)	1(2)	0(0)	1(3)	0(0)	0(0)
Dislocation	3 (3)	1(2)	2(3)	0(0)	0(0)	1(2)	2(6)
Sprain	12 (10)	8(15)	4(6)	0(0)	3(8)	4(10)	5(14)
Ligament injury	9 (7)	2(4)	7(11)	0(0)	1(3)	4(10)	4(11)
Lesion of meniscus or cartilage	7 (6)	4(7)	3(5)	0(0)	2(5)	2(5)	3(9)
Muscle rupture/tear	9 (8)	5(9)	4(6)	2(33)	3(8)	2(5)	2(6)
Muscle strain	32 (29)	11(20)	21(33)	2(33)	10(27)	12(29)	8(23)
Tendon injury/strain	20 (18)	15(27)	5(8)	2(33)	6(16)	8(20)	4(11)
Tendinitis/bursitis	18 (16)	8(15)	10(16)	0(0)	8(22)	6(15)	4(11)
Contusion/hematoma	3 (3)	1(2)	2(3)	0(0)	1(3)	2(5)	1(3)
Nerve injury	3 (3)	0(0)	3(5)	0(0)	2(5)	0(0)	1(3)
During fitness testing (n = 56 injuries)							
Concussion	1 (2)	0(0)	1(3)	0(0)	1(4)	0(0)	0(0)
Dizziness	2 (4)	0(0)	2(6)	0(0)	1(4)	1(6)	0(0)
Fracture	1 (2)	1(5)	0(0)	0(0)	1(4)	0(0)	0(0)
Sprain	1 (2)	1(5)	0(0)	0(0)	0(0)	0(0)	1(8)
Lesion of meniscus or cartilage	2 (4)	1(5)	1(3)	0(0)	2(8)	0(0)	0(0)
Muscle rupture/tear	3 (5)	1(5)	2(6)	0(0)	1(4)	2(12)	0(0)
Muscle strain	33 (59)	13(59)	20(59)	3(100)	12(50)	11(65)	7(58)
Tendon injury/strain	3 (5)	2(9)	1(3)	0(0)	2(8)	1(6)	0(0)
Tendinitis/bursitis	6 (11)	1(5)	5(15)	0(0)	4(17)	0(0)	2(17)
Contusion/hematoma	2 (4)	1(5)	1(3)	0(0)	0(0)	1(6)	1(8)
Nerve injury	2 (4)	1(5)	1(3)	0(0)	0(0)	1(6)	1(8)

The injury incidence per 1000 hours shows that there was no evident difference between the MRs and ARs. Among the MRs, the lowest rate was found for the 3^rd^ league, while for the ARs, the lowest prevalence was found among the UEFA referees (Table 7).

**Table 7:** Number of injuries per 1000 hours of match during the last year

	**Total**	**UEFA**	**1**^**st **^**league**	**2**^**nd **^**league**	**3**^**rd **^**league**
	**Mean (95% CI)**	**Mean (95% CI)**	**Mean (95% CI)**	**Mean (95% CI)**	**Mean (95% CI)**
Match referees	5.29 (2.23–8.30)	3.85 (-0.93–8.33)	12.21 (-0.71–25.09)	6.24 (1.48–10.99)	1.90 (0.47–3.34)
Assistant referees	4.58 (2.63–6.54)	2.01 (-3.72–6.52)	8.37 (3.27–13.46)	5.42 (-0.54–11.39)	2.11 (0.79–3.43)
TOTAL	4.92 (3.17–6.69)	3.33 (-0.44–7.11)	9.90 (4.14–15.66)	5.89 (2.33–9.45)	2.00 (1.05–2.96)

## Discussion

The Croatian soccer referees included in this study were somewhat younger than those previously studied [9,14,20,27], but this is in concordance with the recent UEFA suggestions regarding the need for decreasing the referees’ ages at all competitive levels. In addition, our subjects were taller and had a higher BM than Chilean [7] and Spanish referees [8], while they had higher BM and BMI values than Swiss referees [28].

The rate of approximately 5 injuries per 1000 match hours is similar to those previously reported in retrospective studies performed for Swiss referees (1.6 and 6.8 injuries/1000 hours for AR and MR, respectively) [27]. Meanwhile, prospective investigations show a much higher incidence of injury (up to 20 injuries per 1000 hours of refereeing) [25,30], which is explained by the recall-bias [36]. Our result of 1–2 injuries/1000 hours among UEFA referees is similar to the retrospective data reported for top-level international referees [25].

In the following section, we will focus on the issues that have not been extensively examined in previous studies. These issues mainly relate to (a) the differences between the type and location of injuries occurring during fitness testing and games, (b) the types of injuries and the consequences of those injuries in relation to the level of refereeing, and (c) the comparisons between the main and assistant referees with regard to the type and location of the injuries.

### Fitness testing injuries vs. game injuries

The injury rate of 29% during the last year is similar to rates that have been previously reported for Swiss referees [28]. One of every six subjects suffered from an injury during fitness testing, and therefore, although it is performed in a “controlled environment” (e.g., the tests are known in advance, there are no tests with quick changes of direction, tests are performed on a well-prepared field), fitness testing should be considered potentially dangerous in terms of injury risk. It is especially interesting to compare the number of tests per year (i.e., 4) and the average number of games per year (i.e., 15), which leads us to conclude that physical fitness testing carries a higher risk of injury among soccer referees than match refereeing. There are several possible explanations for these findings. First, participation and successful achievement in fitness testing are the main prerequisites for match refereeing. Therefore, referees are highly concerned about their achievement at every examination. During a game, the referee can “tailor” his method of refereeing and thereby pace the physical demands of refereeing, whereas throughout testing, standards for both tests done throughout fitness testing must be achieved. Most likely, a lack of fitness level and poor preparation are the main risk factors for injury occurrence during fitness testing. This is indirectly demonstrated by the fact that the UEFA referees, who must achieve the highest fitness standards, were less commonly injured during physical fitness testing (i.e., none of the UEFA MRs reported injuries that occurred during fitness testing).

The lower leg region (calf and tendon, represents 31% of all injuries) and the upper leg region (quadriceps and hamstrings, represents 26%) were equally injured during physical fitness testing. During games, however, almost 50% of all injuries were lower leg injuries. The main reason for the dissimilarity between games and fitness testing with regard to the location of injuries is based on the types of activities in these two events. Previous studies noted that soccer referees sprint (run faster than 18 km/h) approximately 1 km in each half of the game [14,15]. Given that one of the fitness tests is completed at maximum running speed, the running intensity is much higher during fitness testing. The upper leg (quadriceps and hamstring) is biomechanically and anatomically more challenged during sprinting than during lower-intensity running [37]. It is therefore logical that studies have found that the quadriceps and hamstring regions are more commonly injured among sprinters than among middle- and long-distance runners [38,39]. This directly explains the higher rate of injury to the upper leg region during fitness tests than during match refereeing. The same logic applies when explaining the relatively higher injury rate for the groin area during physical fitness testing. Although not reported among soccer referees, this problem has been reported in soccer players where more intensive sprinting was related to a higher risk of groin injury in [40].

In contrast to the upper leg and groin area, which are more commonly injured during fitness testing, the knee is injured twice as often during games compared with during fitness testing, which is most likely a result of the characteristic movement patterns during the game. Throughout a soccer match, referees perform numerous changes of direction, some of which are executed during high-intensity runs. In contrast, during fitness testing, such risky movement patterns are not as common, which places the referees at a lower risk for knee injury. Furthermore, throughout testing, the referees focus exclusively on the fitness task, while during a match, they must focus on the game. This focus can place the referees at risk of inappropriate movement patterns, such as a spin turn [41], which consequently increases the possibility of knee injuries. The risk is amplified during the game because refereeing is performed in soccer boots (during the spin-turn the foot is “locked” in the ground), and this is not the case during fitness testing.

### Prevalence and consequences of injuries: are there any differences between the various levels of refereeing?

There is an obvious increase in injuries concurrent with an increase in the referee level. The highest prevalence of injuries is found in referees involved in the 1^st^ league, and the prevalence decreases at lower levels, with the lowest prevalence observed for the 3^rd^ league referees. However, among UEFA referees, the injury rate is somewhat lower, although these referees regularly participate in the national 1^st^ league competitions. Previous studies [27] reported a trend similar to that found in our study. The highest prevalence of match injuries was noted for the highest competitive referee level (5.54 injuries per 1000 match hours). The prevalence rate was lower for the lower level competitions (0.59, 2.57 and 1.97 injuries per 1000 match hours for junior, low-level amateur and high-level amateur referees, respectively). It has been suggested [27,42] that one of the risk factors for injury is the age of the subjects. The lowest level referees, in our case, the 3^rd^ national league, are the youngest. Another explanation for the higher injury rates for higher-level referees may be found in the more advanced level of play and game dynamics, which increases with competitive level [5,6]. However, as stated before, this trend of higher injury rates for higher competitive levels is not evident among the top-level referees (UEFA referees). Even more intriguing is that the UEFA referees are the oldest of all referees studied. There are several possible reasons for their low injury rate. First, the injury rate is one of the selective factors for advancement in refereeing. All soccer referees begin their career at low-level competitions, and their progress depends on the quality of their performance. Those referees who are less frequently injured are more likely to be recognized as successful, which leads to their promotion to higher-level competitions. Most likely even more important is the fact that the UEFA referees are the most strictly monitored for their fitness and training status and fitness achievements [21]. They are obligated to provide a weekly/monthly report of their training throughout the season to the UEFA authorities. This report includes not only subjective reports but also precise and detailed analyses of the overall training volume and intensity and includes heart rate monitoring diaries. Furthermore, their training is prescribed and monitored by UEFA experts. Apart from the already proven superior fitness status [21], such a supervised exercise regime almost certainly places the referees at a lower risk of injury. This explanation is indirectly proven by the fact that none of the UEFA MRs reported any injuries during physical fitness testing, which is a clear indicator of their excellent physical readiness and overall fitness status.

### Comparison between the main and assistant referees with regard to the type and location of injuries

The injury rates of the Croatian soccer referees are similar to those previously reported for Swiss referees (29% of the subjects were injured during the last 12 months, with approximately 5 injuries per 1000 hours) [28]. However, the Swiss study found a higher percentage of injured MRs (36%) compared with ARs (20%), most likely because the authors sampled only top-division referees.

The ratio between the MRs and ARs for injured ankles is approximately 8 to 1, which is explainable by knowing the differences in the MR and AR activities during the game. The movement of the ARs is characterized by short, intense sprints of 5 to 40 meters, interrupted by relatively long, low-intensity periods [21,43]. The MRs sprint at a high intensity over a longer distance than the ARs (up to 80 meters). Additionally, the ARs are placed outside of the official playing field, where the consistency and quality of the turf is far better than within the playing field, where the MRs are positioned. Surface quality has been recognized as an important factor in sports injury rates [44]. These factors (surface quality and differences in the sprint distance) most likely also contribute to the higher prevalence of Achilles tendon injuries among MRs.

Our findings are in accordance with previous studies [25,26] that noted a higher prevalence of lower back injuries among ARs than among MRs. Although not studied in detail in the questionnaire, which was based on previous studies relating lower-back pain to the advanced age of the subjects [45], we believe that the problem is age-related (i.e., ARs are older than MRs). This is supported by the fact that lower-back problems are more prevalent in ARs from the qualitative groups in which assistants are significantly older than their match peers (e.g., 1^st^ and 3^rd^ national leagues). Although not frequent, shoulder injuries are noted exclusively among ARs, and this is almost certainly related to the ARs’ use of flags.

### Study limitations

There are several limitations of the study. First, the study is retrospective in nature. Consequently, the data should be assessed with regard to their possible low reliability. For example, the subjects may have forgotten some previous injuries and musculoskeletal disorders or the consequences of these injuries [36]. However, prior to the testing, we performed a pilot study in which the reliability of the questionnaire was shown to be appropriate (see Methods for more details). Moreover, the study included subjects from only one country, and therefore, the data are of questionable generalizability. However, the sample was relatively large and included more than 90% of all national level referees from the country. In addition, the response rate was high (more than 95%), which is one of the most important factors when studying health-related issues on a self-reported basis [46,47]. Additionally, the investigation was based on self-reports, and the subjects might not have told the truth if they felt uncomfortable. However, we believe that the anonymity and design of the study (e.g., testing in large groups with no pressure on the examinees) decreased this possibility. The study was approved by the highest national soccer organization (Croatian Football Federation) and was therefore officially accepted. The testing was performed by the first author, who is a 1^st^ league MR and a UEFA additional assistant and is therefore an “insider”. This fact almost certainly had a positive effect on the honesty of the examinees, as the examiner was familiar with the problems, and therefore, the subjects had no reason to be untruthful [35,47]. Finally, and with regard to previous studies we must acknowledge that significant limitation should be observed in the fact that we studied only injuries, and did not report complaints, which almost certainly must be emphasized in forthcoming studies. Despite the study’s limitations, we believe that the results (although not the final word on this topic) contribute to knowledge in the field.

## Conclusion

At the national refereeing levels, there is an increase in injuries concurrent with an increased competitive level. However, the low prevalence of injuries among UEFA level referees may be explained by their greater physical fitness and by officially prescribed and supervised UEFA training, overall professionalism, and regular medical assistance.

Although fitness testing is performed in a controlled environment, testing should be considered to be a risk factor for injuries. The differences between match injuries and fitness testing injuries may be due to the characteristics of activities during matches and fitness tests (e.g., maximal sprints are more common during testing).

The unevenness in the playing field together with frequent quick changes of direction are most likely the main contributors to ankle injuries among MRs. The ARs are older, which most likely influences the prevalence of lower back problems among ARs.

In the future, we suggest using a prospective design to provide a deeper insight into the health-related problems and consequences of soccer refereeing.

## Abbreviations

MR: Match referees; AR: Assistant referees; FIFA: Fédération Internationale de Football Association; UEFA: Union of European Football Associations; ANOVA: Analysis of variance.

## Competing interests

The authors declare that they have no competing interests.

## Authors’ contributions

GG designed the study, tested the subjects, performed the statistical analysis and discussed the data; KI designed the study and drafted the manuscript; MO reviewed the previous studies and discussed the results; BN reviewed the previous studies and discussed the data; and DS leaded the project reviewed the statistics, discussed the data and drafted the manuscript. All authors have read and approved the final version.

## Pre-publication history

The pre-publication history for this paper can be accessed here:

http://www.biomedcentral.com/1471-2474/14/88/prepub

## Supplementary Material

Additional file 1 Questionnaire. Click here for file

Additional file 2 Multimedia presentation of the project. Click here for file

## References

[B1] Lindner AM Hawkins DN Globalization, Culture Wars, and Attitudes toward Soccer in America: An Empirical Assessment of How Soccer Explains the World Sociol Quart 2012 53 1 68 91 10.1111/j.1533-8525.2011.01226.x

[B2] Brandes L Franck E Nuesch S Local heroes and superstars-An empirical analysis of star attraction in German soccer J Sport Econ 2008 9 3 266 286

[B3] Vaeyens R Lenoir M Williams AM Philippaerts RM Talent identification and development programmes in sport-Current models and future directions Sports Med 2008 38 9 703 714 10.2165/00007256-200838090-00001 18712939

[B4] Rowe D Gilmour C Global sport: Where Wembley Way meets Bollywood Boulevard Continuum-J Media Cu 2009 23 2 171 182 10.1080/10304310802710512

[B5] Mohr M Krustrup P Andersson H Kirkendal D Bangsbo J Match activities of elite women soccer players at different performance levels J Strength Cond Res 2008 22 2 341 349 10.1519/JSC.0b013e318165fef6 18550946

[B6] Mohr M Krustrup P Bangsbo J Match performance of high-standard soccer players with special reference to development of fatigue J Sports Sci 2003 21 7 519 528 10.1080/0264041031000071182 12848386

[B7] Vargas GEF da Silva AI Arruda M Anthropometric Profile and Physical Fitness of the Professional Referees Chilean Soccer Int J Morphol 2008 26 4 897 904

[B8] Caballero JAR Ojeda EB Garcia-Aranda JM Mallo J Helsen W Sarmiento S Garcia-Manso JM Valdivielso MEN Echocardiographic study of structure and functional cardiac profile of football referees J Sport Med Phys Fit 2011 51 4 633 638 22212266

[B9] Rontoyannis GP Stalikas A Sarros G Vlastaris A Medical, morphological and functional aspects of Greek football referees J Sports Med Phys Fitness 1998 38 3 208 214 9830827

[B10] Dottavio S Castagna C Analysis of match activities in elite soccer referees during actual match play J Strength Cond Res 2001 15 2 167 171 11710400

[B11] FIFA Big Count [http://www.fifa.com/worldfootball/bigcount/]

[B12] Mallo J Veiga S Lopez De Subijana C Navarro E Activity profile of top-class female soccer refereeing in relation to the position of the ball J Sci Med Sport 2010 13 1 129 132 10.1016/j.jsams.2008.09.006 19084474

[B13] Weston M Castagna C Impellizzeri FM Rampinini E Abt G Analysis of physical match performance in English Premier League soccer referees with particular reference to first half and player work rates J Sci Med Sport 2007 10 6 390 397 10.1016/j.jsams.2006.09.001 17126077

[B14] Castagna C Abt G D'Ottavio S Activity profile of international-level soccer referees during competitive matches J Strength Cond Res 2004 18 3 486 490 15320668 10.1519/1533-4287(2004)18<486:APOISR>2.0.CO;2

[B15] Castagna C Abt G Intermatch variation of match activity in elite Italian soccer referees J Strength Cond Res 2003 17 2 388 392 12741883 10.1519/1533-4287(2003)017<0388:ivomai>2.0.co;2

[B16] Castagna C Abt G D'Ottavio S The relationship between selected blood lactate thresholds and match performance in elite soccer referees J Strength Cond Res 2002 16 4 623 627 12423196

[B17] Castagna C Abt G D'Ottavio S Relation between fitness tests and match performance in elite Italian soccer referees J Strength Cond Res 2002 16 2 231 235 11991775

[B18] Weston M Gregson W Castagna C Breivik S Impellizzeri FM Lovell RJ Changes in a top-level soccer referee's training, match activities, and physiology over an 8-year period: a case study Int J Sports Physiol Perform 2011 6 2 281 286 21725113 10.1123/ijspp.6.2.281

[B19] Weston M Castagna C Helsen W Impellizzeri F Relationships among field-test measures and physical match performance in elite-standard soccer referees J Sports Sci 2009 27 11 1177 1184 10.1080/02640410903110982 19724966

[B20] Reilly T Gregson W Special populations: the referee and assistant referee J Sports Sci 2006 24 7 795 801 10.1080/02640410500483089 16766507

[B21] Bartha C Petridis L Hamar P Puhl S Castagna C Fitness test results of Hungarian and international-level soccer referees and assistants J Strength Cond Res 2009 23 1 121 126 10.1519/JSC.0b013e31818ebb84 19125100

[B22] Casajus JA Castagna C Aerobic fitness and field test performance in elite Spanish soccer referees of different ages J Sci Med Sport 2007 10 6 382 389 10.1016/j.jsams.2006.08.004 17116419

[B23] Castagna C Abt G D'Ottavio S Physiological aspects of soccer refereeing performance and training Sports Med 2007 37 7 625 646 10.2165/00007256-200737070-00006 17595157

[B24] Fauno P Kalund S Andreasen I Jorgensen U Soreness in lower extremities and back is reduced by use of shock absorbing heel inserts Int J Sports Med 1993 14 5 288 290 10.1055/s-2007-1021179 8365838

[B25] Bizzini M Junge A Bahr R Helsen W Dvorak J Injuries and musculoskeletal complaints in referees and assistant referees selected for the 2006 FIFA World Cup: retrospective and prospective survey Br J Sports Med 2009 43 7 490 497 10.1136/bjsm.2008.048314 18603580

[B26] Bizzini M Junge A Bahr R Dvorak J Female soccer referees selected for the FIFA Women's World Cup 2007: survey of injuries and musculoskeletal problems Br J Sports Med 2009 43 12 936 942 10.1136/bjsm.2008.051318 18927163

[B27] Bizzini M Junge A Bahr R Dvorak J Injuries of football referees: a representative survey of Swiss referees officiating at all levels of play Scand J Med Sci Sports 2011 21 1 42 47 10.1111/j.1600-0838.2009.01003.x 19883383

[B28] Bizzini M Junge A Bahr R Dvorak J Injuries and musculoskeletal complaints in referees-a complete survey in the top divisions of the swiss football league Clin J Sport Med 2009 19 2 95 100 10.1097/JSM.0b013e3181948ad4 19451762

[B29] Weston M Castagna C Impellizzeri FM Bizzini M Williams AM Gregson W Science and medicine applied to soccer refereeing: an update Sports Med 2012 42 7 615 631 10.2165/11632360-000000000-00000 22640237

[B30] Wilson F Byrne A Gissane C A prospective study of injury and activity profile in elite soccer referees and assistant referees Ir Med J 2011 104 10 295 297 22256439

[B31] Zinn C Schofield G Wall C Development of a psychometrically valid and reliable sports nutrition knowledge questionnaire J Sci Med Sport 2005 8 3 346 351 10.1016/S1440-2440(05)80045-3 16248475

[B32] Kucera KL Marshall SW Bell DR DiStefano MJ Goerger CP Oyama S Validity of soccer injury data from the National Collegiate Athletic Association's Injury Surveillance System J Athl Train 2011 46 5 489 499 22488136 10.4085/1062-6050-46.5.489 PMC3418955

[B33] Fuller CW Ekstrand J Junge A Andersen TE Bahr R Dvorak J Hagglund M McCrory P Meeuwisse WH Consensus statement on injury definitions and data collection procedures in studies of football (soccer) injuries Br J Sports Med 2006 40 3 193 201 10.1136/bjsm.2005.025270 16505073 PMC2491990

[B34] Newcombe RG Two-sided confidence intervals for the single proportion: comparison of seven methods Stat Med 1998 17 8 857 872 10.1002/(SICI)1097-0258(19980430)17:8<857::AID-SIM777>3.0.CO;2-E 9595616

[B35] Kondric M Sekulic D Petroczi A Ostojic L Rodek J Ostojic Z Is there a danger for myopia in anti-doping education? Comparative analysis of substance use and misuse in Olympic racket sports calls for a broader approach Subst Abuse Treat Prev Policy 2011 6 27 10.1186/1747-597X-6-27 21988896 PMC3204239

[B36] Junge A Dvorak J Influence of definition and data collection on the incidence of injuries in football Am J Sports Med 2000 28 5 Suppl S40 S46 11032106 10.1177/28.suppl_5.s-40

[B37] Kivi DM Maraj BK Gervais P A kinematic analysis of high-speed treadmill sprinting over a range of velocities Med Sci Sports Exerc 2002 34 4 662 666 10.1097/00005768-200204000-00016 11932576

[B38] Yeung SS Suen AM Yeung EW A prospective cohort study of hamstring injuries in competitive sprinters: preseason muscle imbalance as a possible risk factor Br J Sports Med 2009 43 8 589 594 10.1136/bjsm.2008.056283 19174411

[B39] Askling C Saartok T Thorstensson A Type of acute hamstring strain affects flexibility, strength, and time to return to pre-injury level Br J Sports Med 2006 40 1 40 44 10.1136/bjsm.2005.018879 16371489 PMC2491922

[B40] Emery CA Identifying risk factors for hamstring and groin injuries in sport: a daunting task Clin J Sport Med 2012 22 1 75 77 22228291 10.1097/01.jsm.0000410963.91346.cd

[B41] Wang H Zheng N Knee rotation and loading during spin and step turn Int J Sports Med 2010 31 10 742 746 10.1055/s-0030-1261942 20645235

[B42] Blake C Sherry J Gissane C A survey of referee participation, training and injury in elite gaelic games referees BMC Musculoskelet Disord 2009 10 74 10.1186/1471-2474-10-74 19545401 PMC2713203

[B43] Krustrup P Mohr M Bangsbo J Activity profile and physiological demands of top-class soccer assistant refereeing in relation to training status J Sports Sci 2002 20 11 861 871 10.1080/026404102320761778 12430989

[B44] Wright JM Webner D Playing Field Issues in Sports Medicine Curr Sports Med Rep 2010 9 3 129 133 20463494 10.1249/JSR.0b013e3181df1179

[B45] Hoskins W Pollard H Daff C Odell A Garbutt P McHardy A Hardy K Dragasevic G Low back pain in junior Australian rules football: a cross-sectional survey of elite juniors, non-elite juniors and non-football playing controls BMC Musculoskelet Disord 2010 11 241 10.1186/1471-2474-11-241 20958973 PMC2967511

[B46] Sekulic D Ostojic M Ostojic Z Hajdarevic B Ostojic L Substance abuse prevalence and its relation to scholastic achievement and sport factors: An analysis among adolescents of the Herzegovina-Neretva Canton in Bosnia and Herzegovina BMC Publ Health 2012 12 1 274 10.1186/1471-2458-12-274 PMC3407773 22480230

[B47] Rodek J Sekulic D Kondric M Dietary supplementation and doping-related factors in high-level sailing J Int Soc Sports Nutr 2012 9 1 51 10.1186/1550-2783-9-51 23217197 PMC3536606

