# Supplementary material for: A retrospective survey on injuries in Croatian football/soccer referees
Source: BMC Musculoskelet Disord. 2013 Mar 11;14:88. doi: 10.1186/1471-2474-14-88 (PMC3599725; doi:10.1186/1471-2474-14-88)
Supplement: Additional file 1 — Questionnaire. [file 1471-2474-14-88-S1.doc]

AGE (IN YEARS): _________________

HEIGHT_______ cm WEIGHT________kg BOOT NO. __________

RANK UEFA 1^ST^ DIV 2^ND^ DIV 3^RD^ DIV

DOMINANT SIDE RIGHT LEFT

# HISTORY OF REFEREEING

***It's of great importance to answer sincerely and accurately on following questions because the aim of this study is to improve overall health status. In case of multiple answers, please note multiple answers. Thank you***

First, we would like to know some details about your history of refereeing

1. Are you match referee or assistant referee
2. Match referee
3. Assistant referee
4. When did you receive the first official license for refereeing (Year) _____________
5. When did you receive the national license for refereeing (Year) _____________
6. When did you star to refereeing 2^ND^ division of national football (Year) ____________
7. When did you star to refereeing 1^ST^ division of national football (Year) ____________
8. When did you receive the FIFA license for refereeing (Year) ____________ (FIFA referees only)
9. When did you refereed first international match (Year) ____________ (FIFA referees only)
10. How many national matches did you referee in your career
    1. Match referee _____________
    2. Assistant referee_____________
11. How many international matches did you referee in your career
    1. Match referee _____________
    2. Assistant referee_____________
12. How many international and national matches do you referee on average per year
    1. International____________
    2. National ____________
13. How many international and national matches did you referee in last year
    1. International____________
    2. National ____________
14. What was the maximum of matches per week you refereed (international, national, youth...) ________________

# TRAINING

- - - 1. Have you ever failed on referees Fitness test?

NO YES, how many times __________

- - - 1. On average how many times (hours) per week do you train for refereeing?(note both: how many times and then hours*)*

PRE-SEASON ______(______) SEASON ______(______)

- - - 1. Do you train the entire year or have breaks?

NO BREAKS _______ WEEKS BREAK

- - - 1. Do you receive any advice for the design of your training program from a qualified professional?

NO, I design training program by my self

YES, specify (Fitness coach, athletics coach, football coach…)_________________

- - - 1. What type of activities your training consists of?

Running (outdoor running, athletic track – tartan…)

Specific running (football field – specific exercise)

Playing futsal

Other activities, please specify______________________

- - - 1. How often do you play futsal?

2-3 per week

Once a week

1-2 per month

Few times a year

Never

- - - 1. How often do you play some other sport (tennis, badminton…)?(please specify)_____

2-3 per week

Once a week

1-2 per month

Few times a year

Never

- - - 1. Do you do anything to prevent injury or complaints?

NO YES, STRETCHING YES, MASSAGE YES, other_____________

# INJURIES AND COMPLAINTS DURING YOUR LAST MATCH

1. When was the last match you refereed_______________ (dd/mm/yy)
2. Level of that match?

International

1^st^ division

2^nd^ division

3^rd^ division

Youth

International friendly

1. Did you have any of the following complaints prior, during or after your last match?

Headache NO YES

Neck pain NO YES

Low back pain NO YES

Pain in the hip joint(s) NO RIGHT LEFT

Pain in the groin region(s) NO RIGHT LEFT

Pain in the inner thigh muscles (adductors) NO RIGHT LEFT

Pain in the front thigh muscles (quadriceps) NO RIGHT LEFT

Pain in the back thigh muscles (hamstrings) NO RIGHT LEFT

Pain in the knee joint(s) NO RIGHT LEFT

Pain in the front lower leg NO RIGHT LEFT

Pain in the back lower leg (calf) NO RIGHT LEFT

Pain in the Achilles tendon(s) NO RIGHT LEFT

Pain in the ankle joint(s) NO RIGHT LEFT

Pain in the foot NO RIGHT LEFT

Other (please specify)­­­­­­­­­­­­­­­­____________________________________________________

1. Did you incur an injury during your last match?

NO (please continue with IV.)

YES (please describe injury on following page)

## INJURY (caused by refereeing in your last match)

1. Witch body part was injured?

head / face shoulder hip

neck upper arm groin

sternum / ribs elbow thigh

upper back forearm knee

lower back wrist lower leg

stomach hand Achilles tendon

pelvis / tailbone fingers/thumb ankle

foot/toe

1. What was the type of injury?

concussion (with or with- lesion of meniscus or contusion/bruise/

out loss of consciousness) cartilage haematoma

fracture muscle rupture/strain/ abrasion

other bone injury tear/cramps laceration

dislocation tendon injury/rupture nerve injury

sprain/ligament injury tendinosis/bursitis dental injury

Other injury (please specify)________________________________________

1. Have you had a previous injury of the same type at the same site (recurrence) ?

NO YES (and I was still suffering the complaints)

YES (but I had returned to full participation, since_____)

1. Was the injury caused by overuse or trauma?

Overuse Trauma

1. Did you see a family doctor, a specialists (traumatologist, orthopaedist…) or a physio?

NO YES (family) YES (specialist) YES (physio)

1. How long did you suffer complaints from this injury? ___ days ___ weeks ___ months

until now

1. How long were you absent from regular training? ___ days ___ weeks ___ months

no modification or stop of regular training

1. How long were you absent from refereeing ___ days ___ weeks ___ months
2. When did you incur your first injury caused by refereeing or the training and how? _______ (Year) Training Match

# INJURIES AND COMPLAINTS DURING LAST 12 MONTHS Please specify all injuries

- 1. Have you had any pain, discomfort or complaints caused by refereeing a football match or the training you do preparing for refereeing (last 12 months)

Please indicate whether and to what extent you suffered from any of the following symptoms:

|  | NO | A LITTLE | MODERATE | SEVERELY | VERY SEVERELY |
| --- | --- | --- | --- | --- | --- |
| Headache |  |  |  |  |  |
| Neck pain |  |  |  |  |  |
| Low back pain |  |  |  |  |  |
| Pain in the hip joint(s) |  |  |  |  |  |
| Pain in the groin region(s) |  |  |  |  |  |
| Pain in the front tigh muscles (quadriceps) |  |  |  |  |  |
| Pain in the back tigh muscles (hamstrings) |  |  |  |  |  |
| Pain in the inner tigh muscles (adductors) |  |  |  |  |  |
| Pain in the knee joint(s) |  |  |  |  |  |
| Pain in the Achilles tendon(s) |  |  |  |  |  |
| Pain in the back lower leg (calf) |  |  |  |  |  |
| Pain in the front lower leg |  |  |  |  |  |
| Pain in the ankle joint(s) |  |  |  |  |  |
| Pain in the foot |  |  |  |  |  |
| Other (please specify) |  |  |  |  |  |

1. Did you suffer any injury caused by refereeing a football match or the preparing training during the last 12 months?

NO (please continue with section V.)

YES, how many? __________

## INJURY DURING LAST 12 MONTHS

# Please specify all injuries. In case of multiple injuries, please fill in one page for each injury

1. Witch body part were injured?

head / face shoulder hip

neck upper arm groin

sternum / ribs elbow thigh

upper back forearm knee

lower back wrist lower leg

stomach hand Achilles tendon

pelvis / tailbone fingers/thumb ankle

foot/toe

1. What was the type of injury?

concussion (with or with- lesion of meniscus or contusion/bruise/

out loss of consciousness) cartilage haematoma

fracture muscle rupture/strain/ abrasion

other bone injury tear/cramps laceration

dislocation tendon injury/rupture nerve injury

sprain/ligament injury tendinosis/bursitis dental injury

Other injury (please specify)________________________________________

1. Have you had a previous injury of the same type at the same site (recurrence) ?

NO YES (and I was still suffering the complaints)

YES (but I had returned to full participation, since_____)

1. Was the injury caused by overuse or trauma?

Overuse Trauma

1. Did you see a family doctor, a specialists (traumatologist, orthopaedist…) or a physio?

NO YES (family) YES (specialist) YES (physio)

1. How long did you suffer complaints from this injury? ___ days ___ weeks ___ months

Until now

1. How long were you absent from regular training? ___ days ___ weeks ___ months

no modification or stop of regular training

1. How long were you absent from refereeing ___ days ___ weeks ___ months
2. When did you incur your first injury caused by refereeing or the training and how? _______ (Year) Training Match

# INJURIES AND COMPLAINTS DURING YOUR REFEREEING CAREER

1. Have you had any pain, discomfort or complaints caused by refereeing a football match or the training you do preparing for refereeing during your refereeing career?

Please indicate whether and to what extent you suffered from any of the following symptoms:

|  | NO | A LITTLE | MODERATE | SEVERELY | VERY SEVERELY |
| --- | --- | --- | --- | --- | --- |
| Headache |  |  |  |  |  |
| Neck pain |  |  |  |  |  |
| Low back pain |  |  |  |  |  |
| Pain in the hip joint(s) |  |  |  |  |  |
| Pain in the groin region(s) |  |  |  |  |  |
| Pain in the front tigh muscles (quadriceps) |  |  |  |  |  |
| Pain in the back tigh muscles (hamstrings) |  |  |  |  |  |
| Pain in the inner tigh muscles (adductors) |  |  |  |  |  |
| Pain in the knee joint(s) |  |  |  |  |  |
| Pain in the Achilles tendon(s) |  |  |  |  |  |
| Pain in the back lower leg (calf) |  |  |  |  |  |
| Pain in the front lower leg |  |  |  |  |  |
| Pain in the ankle joint(s) |  |  |  |  |  |
| Pain in the foot |  |  |  |  |  |
| Other (please specify) |  |  |  |  |  |

1. Did you suffer any injury caused by refereeing a football match or the preparing training during your refereeing career?

NO (please continue with section VI.)

YES, how many? __________

## INJURY DURING REFEREEING CAREER

# Please specify all injuries. In case of multiple injuries, please fill in one page for each injury

1. Witch body part were injured?

head / face shoulder hip

neck upper arm groin

sternum / ribs elbow thigh

upper back forearm knee

lower back wrist lower leg

stomach hand Achilles tendon

pelvis / tailbone fingers/thumb ankle

foot/toe

1. What was the type of injury?

concussion (with or with- lesion of meniscus or contusion/bruise/

out loss of consciousness) cartilage haematoma

fracture muscle rupture/strain/ abrasion

other bone injury tear/cramps laceration

dislocation tendon injury/rupture nerve injury

sprain/ligament injury tendinosis/bursitis dental injury

Other injury (please specify)________________________________________

1. Have you had a previous injury of the same type at the same site (recurrence) ?

NO YES (and I was still suffering the complaints)

YES (but I had returned to full participation, since_____)

1. Was the injury caused by overuse or trauma?

Overuse Trauma

1. Did you see a family doctor, a specialists (traumatologist, orthopaedist…) or a physio?

NO YES (family) YES (specialist) YES (physio)

1. How long did you suffer complaints from this injury? ___ days ___ weeks ___ months

until now

1. How long were you absent from regular training? ___ days ___ weeks ___ months

no modification or stop of regular training

1. How long were you absent from refereeing ___ days ___ weeks ___ months
2. When did you incur your first injury caused by refereeing or the training and how? _______ (Year) Training Match

# INJURIES THAT OCCURRED DURING FITNESS TESTING

# Please specify all injuries. In case of multiple injuries, please fill in one page for each injury

1. Did you suffer any injury during fitness tests or training for it?

NO YES, how many _____

1. Witch body part were injured?

head / face shoulder hip

neck upper arm groin

sternum / ribs elbow thigh

upper back forearm knee

lower back wrist lower leg

stomach hand Achilles tendon

pelvis / tailbone fingers/thumb ankle

foot/toe

1. What was the type of injury?

concussion (with or with- lesion of meniscus or contusion/bruise/

out loss of consciousness) cartilage haematoma

fracture muscle rupture/strain/ abrasion

other bone injury tear/cramps laceration

dislocation tendon injury/rupture nerve injury

sprain/ligament injury tendinosis/bursitis dental injury

Other injury (please specify)________________________________________

1. Have you had a previous injury of the same type at the same site (recurrence) ?

NO YES (and I was still suffering the complaints)

YES (but I had returned to full participation, since_____)

1. Was the injury caused by overuse or trauma?

Overuse Trauma

1. Did you see a family doctor, a specialists (traumatologist, orthopaedist…) or a physio?

NO YES (family) YES (specialist) YES (physio)

1. How long did you suffer complaints from this injury? ___ days ___ weeks ___ months

until now

1. How long were you absent from regular training? ___ days ___ weeks ___ months

no modification or stop of regular training

1. How long were you absent from refereeing ___ days ___ weeks ___ months
