# Supplementary material for: A retrospective survey on injuries in Croatian football/soccer referees
Source: BMC Musculoskelet Disord. 2013 Mar 11;14:88. doi: 10.1186/1471-2474-14-88 (PMC3599725; doi:10.1186/1471-2474-14-88)
Supplement: Additional file 2 — Multimedia presentation of the project. [file 1471-2474-14-88-S2.ppt]

## Slide 1
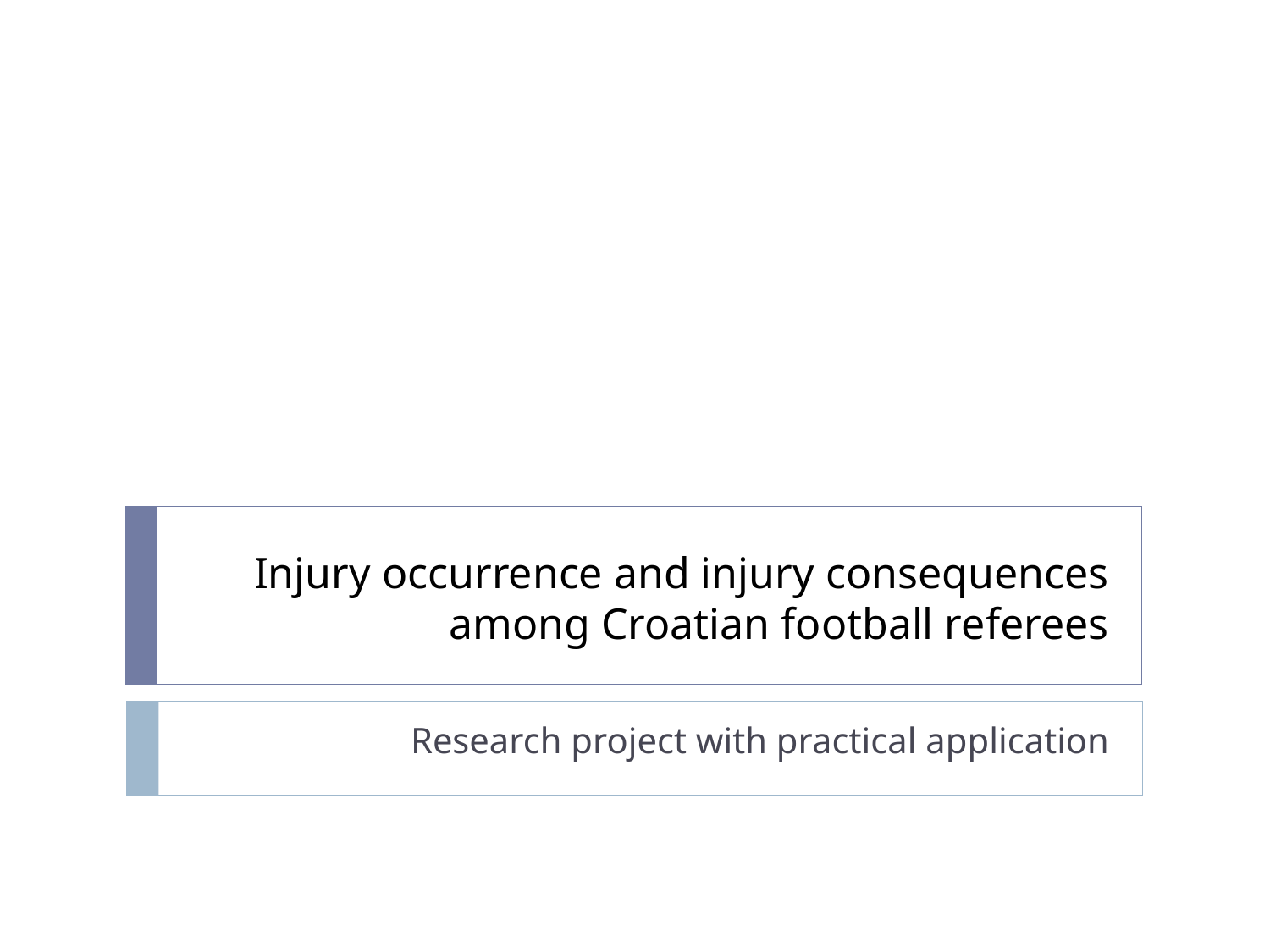

# Injury occurrence and injury consequences among Croatian football referees
Research project with practical application

## Slide 2
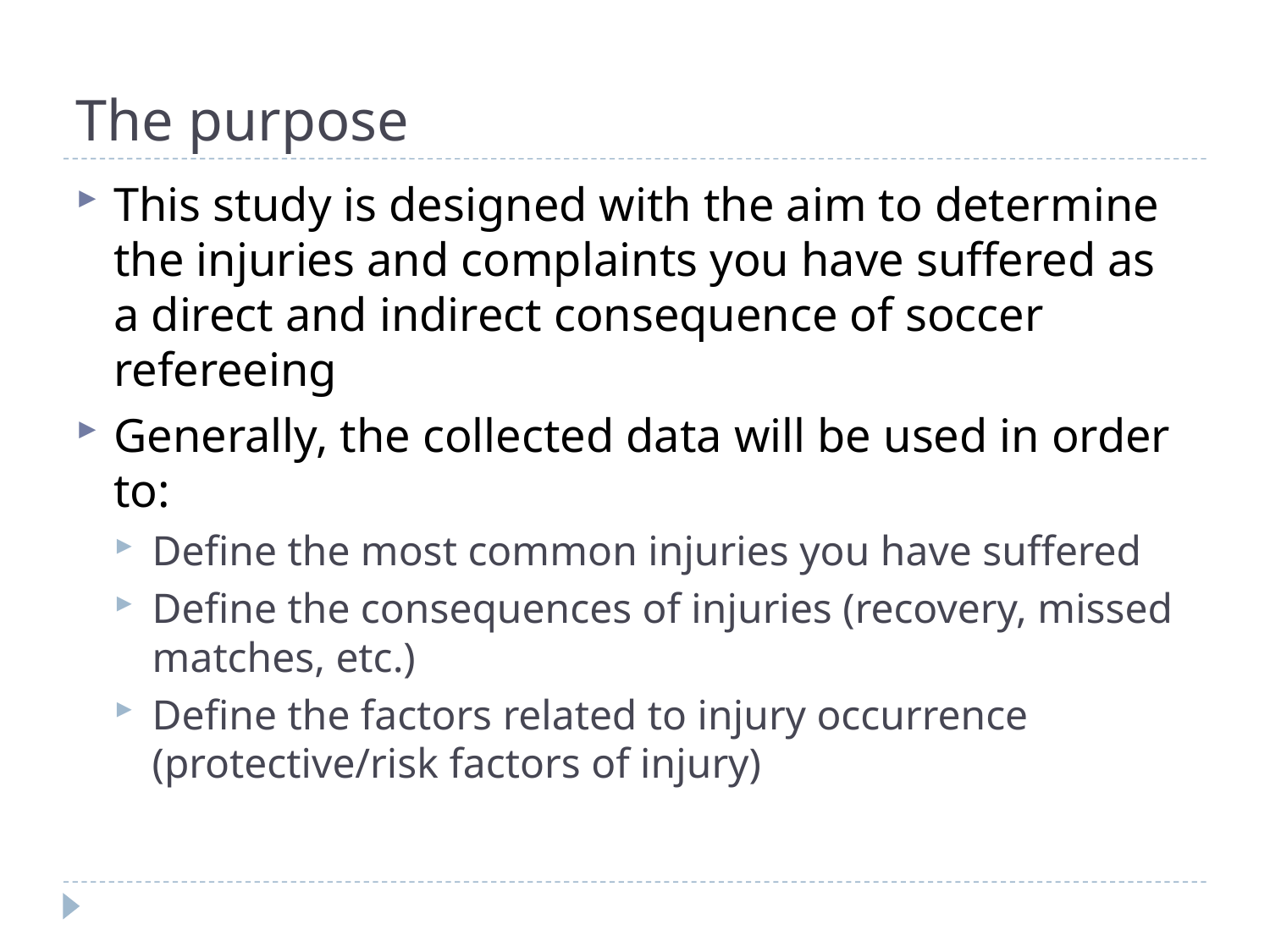

# The purpose
This study is designed with the aim to determine the injuries and complaints you have suffered as a direct and indirect consequence of soccer refereeing
Generally, the collected data will be used in order to:
Define the most common injuries you have suffered
Define the consequences of injuries (recovery, missed matches, etc.)
Define the factors related to injury occurrence (protective/risk factors of injury)

## Slide 3
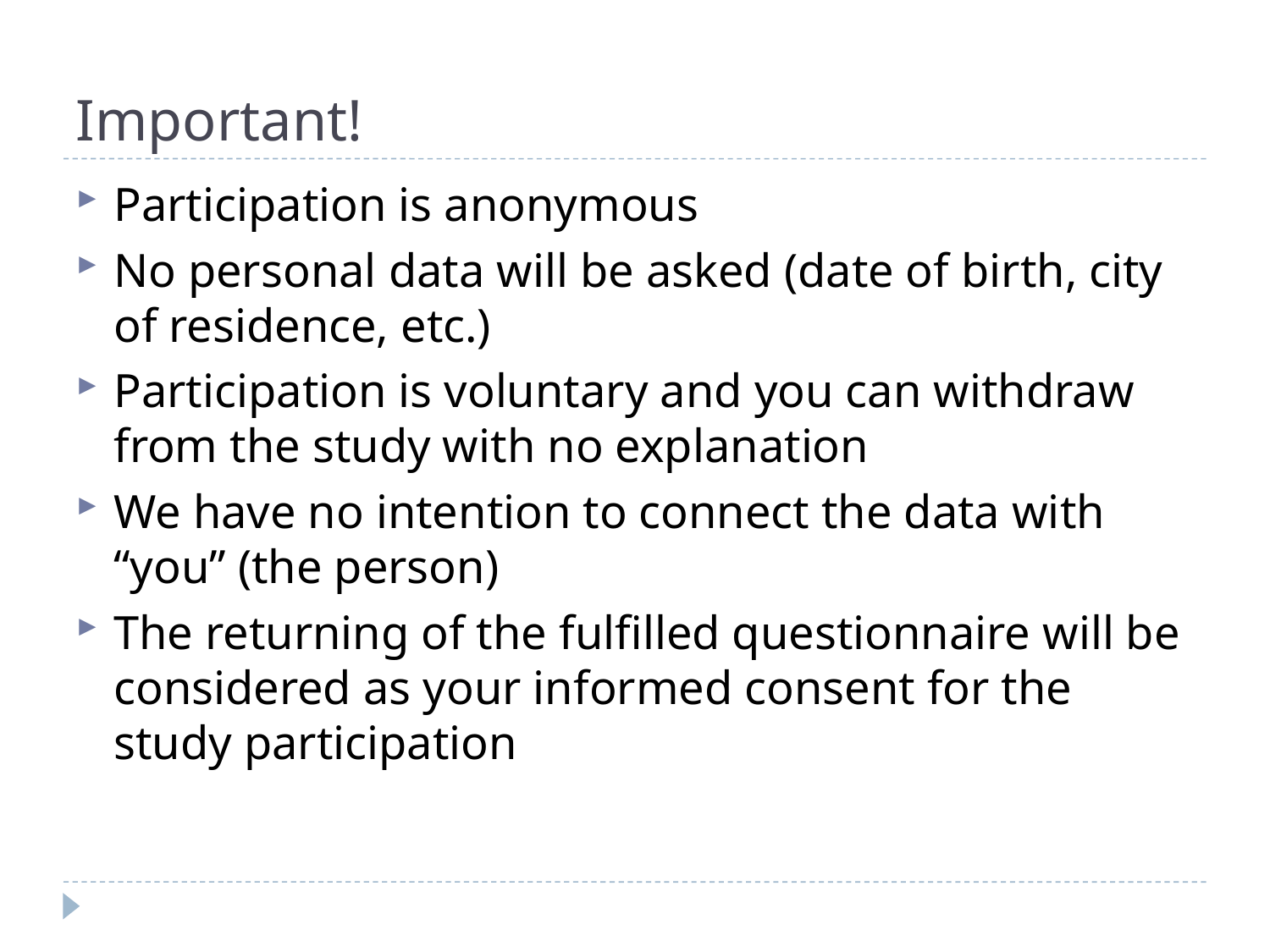

# Important!
Participation is anonymous
No personal data will be asked (date of birth, city of residence, etc.)
Participation is voluntary and you can withdraw from the study with no explanation
We have no intention to connect the data with “you” (the person)
The returning of the fulfilled questionnaire will be considered as your informed consent for the study participation

## Slide 4
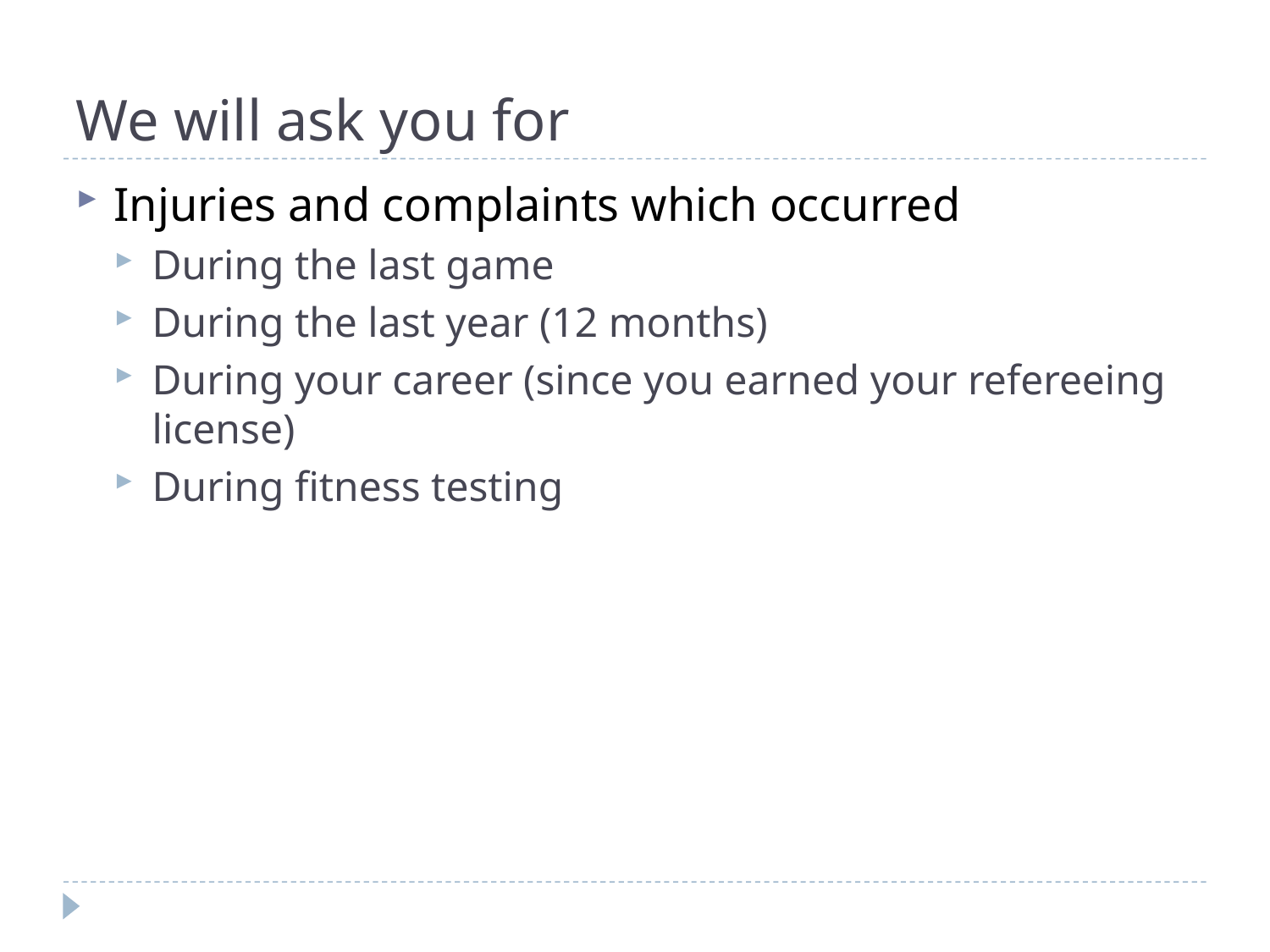

# We will ask you for
Injuries and complaints which occurred
During the last game
During the last year (12 months)
During your career (since you earned your refereeing license)
During fitness testing

## Slide 5
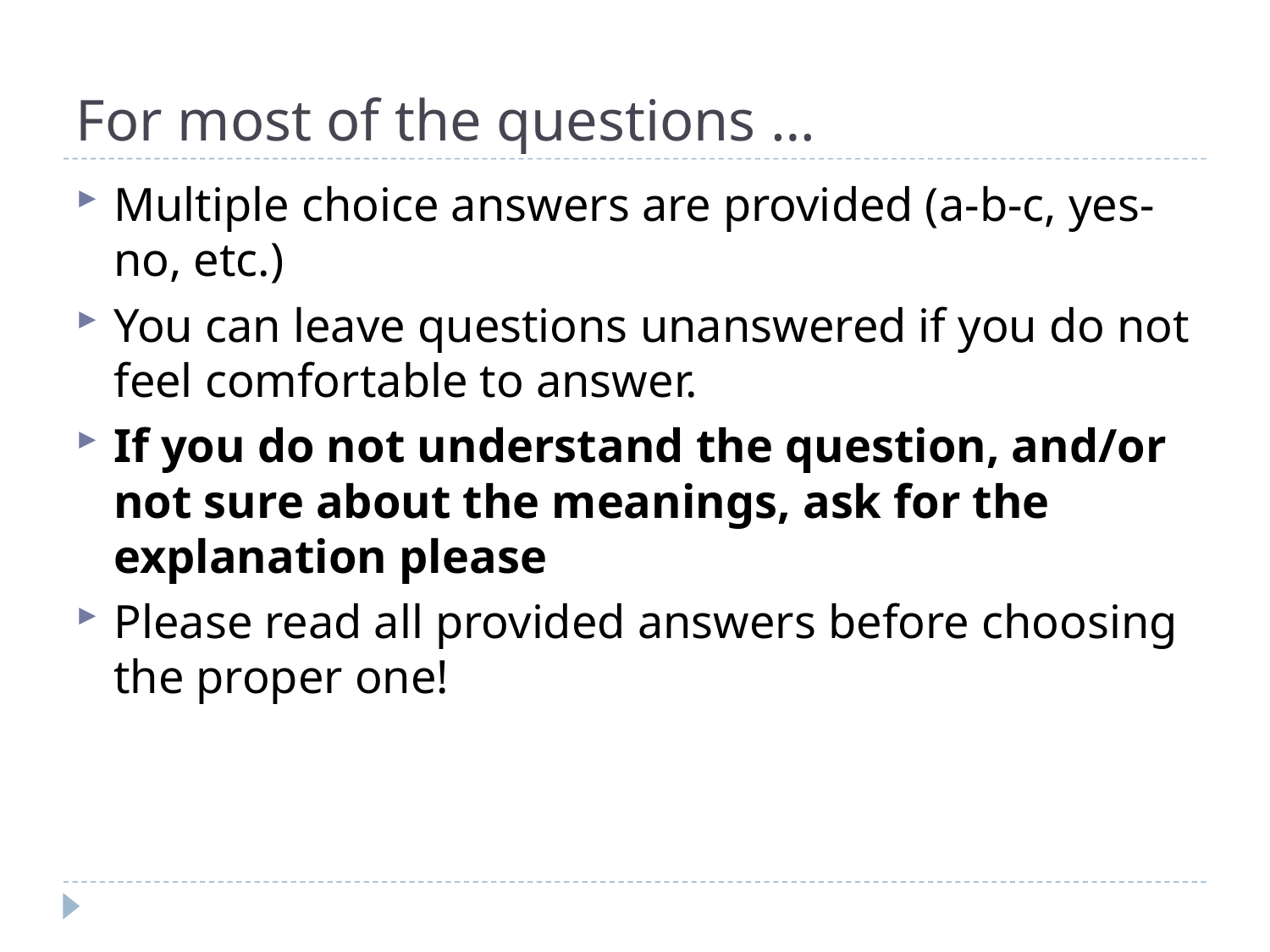

# For most of the questions …
Multiple choice answers are provided (a-b-c, yes-no, etc.)
You can leave questions unanswered if you do not feel comfortable to answer.
If you do not understand the question, and/or not sure about the meanings, ask for the explanation please
Please read all provided answers before choosing the proper one!

## Slide 6
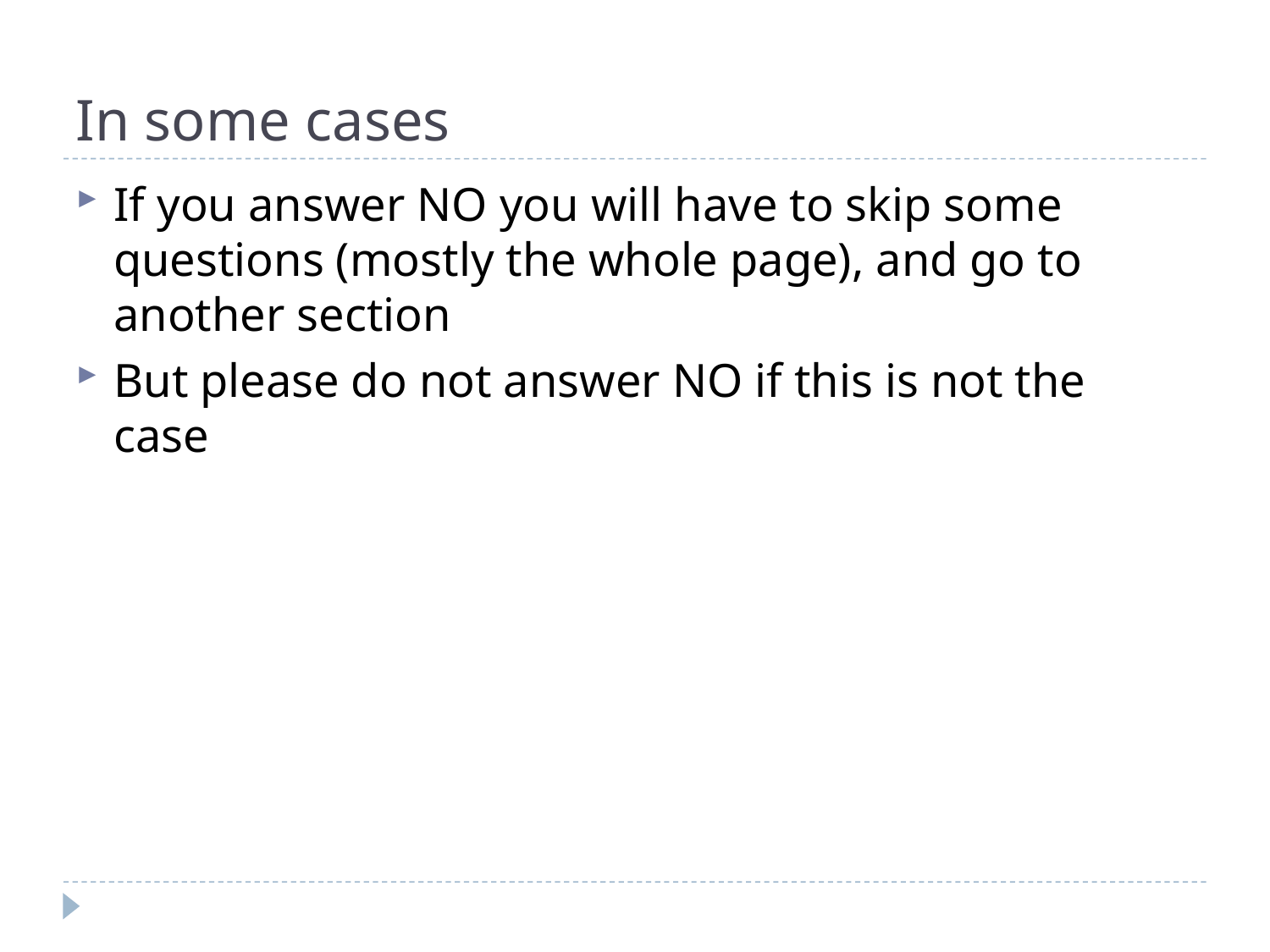

# In some cases
If you answer NO you will have to skip some questions (mostly the whole page), and go to another section
But please do not answer NO if this is not the case

## Slide 7
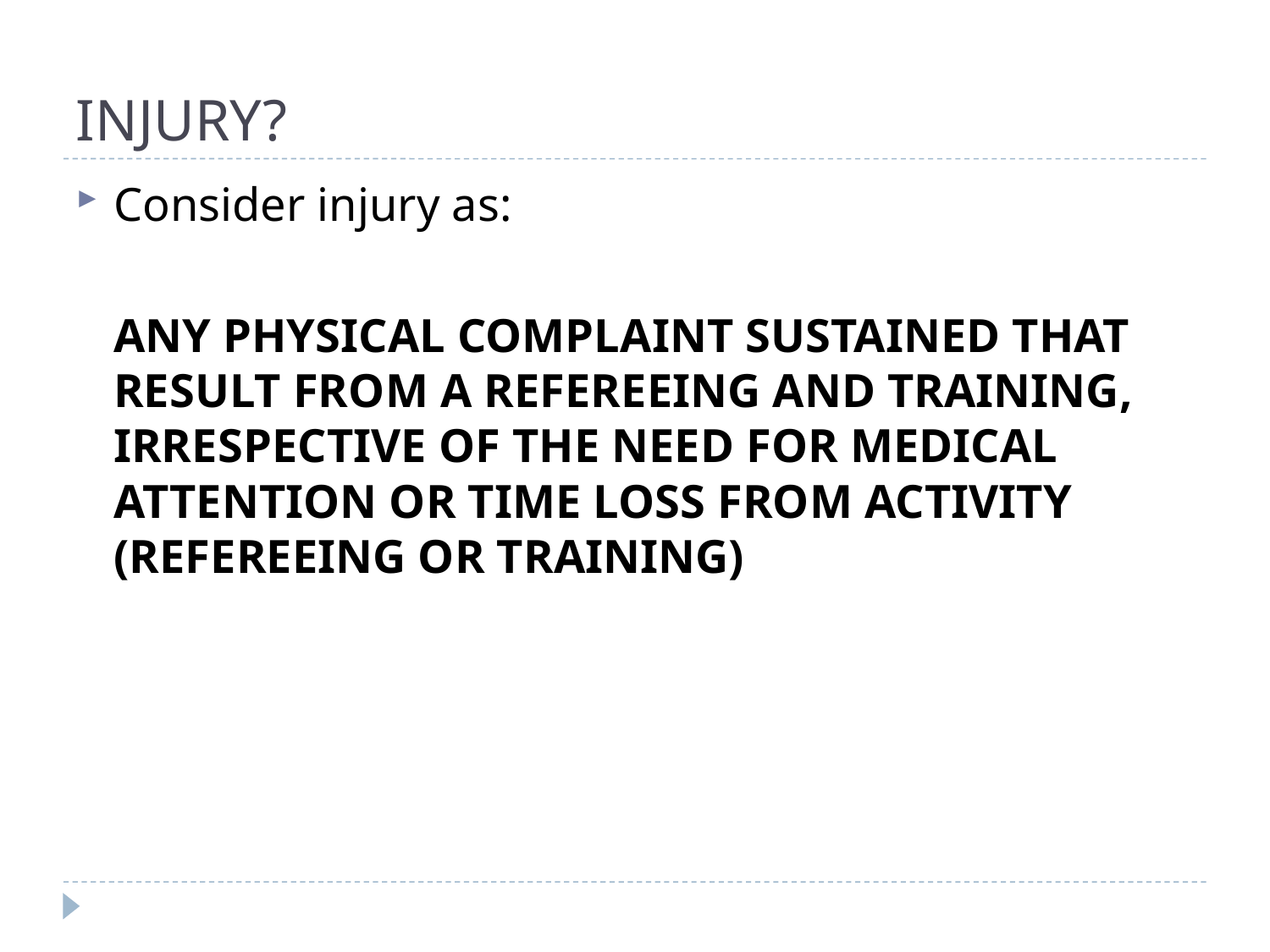

# INJURY?
Consider injury as:
	ANY PHYSICAL COMPLAINT SUSTAINED THAT RESULT FROM A REFEREEING AND TRAINING, IRRESPECTIVE OF THE NEED FOR MEDICAL ATTENTION OR TIME LOSS FROM ACTIVITY (REFEREEING OR TRAINING)

## Slide 8
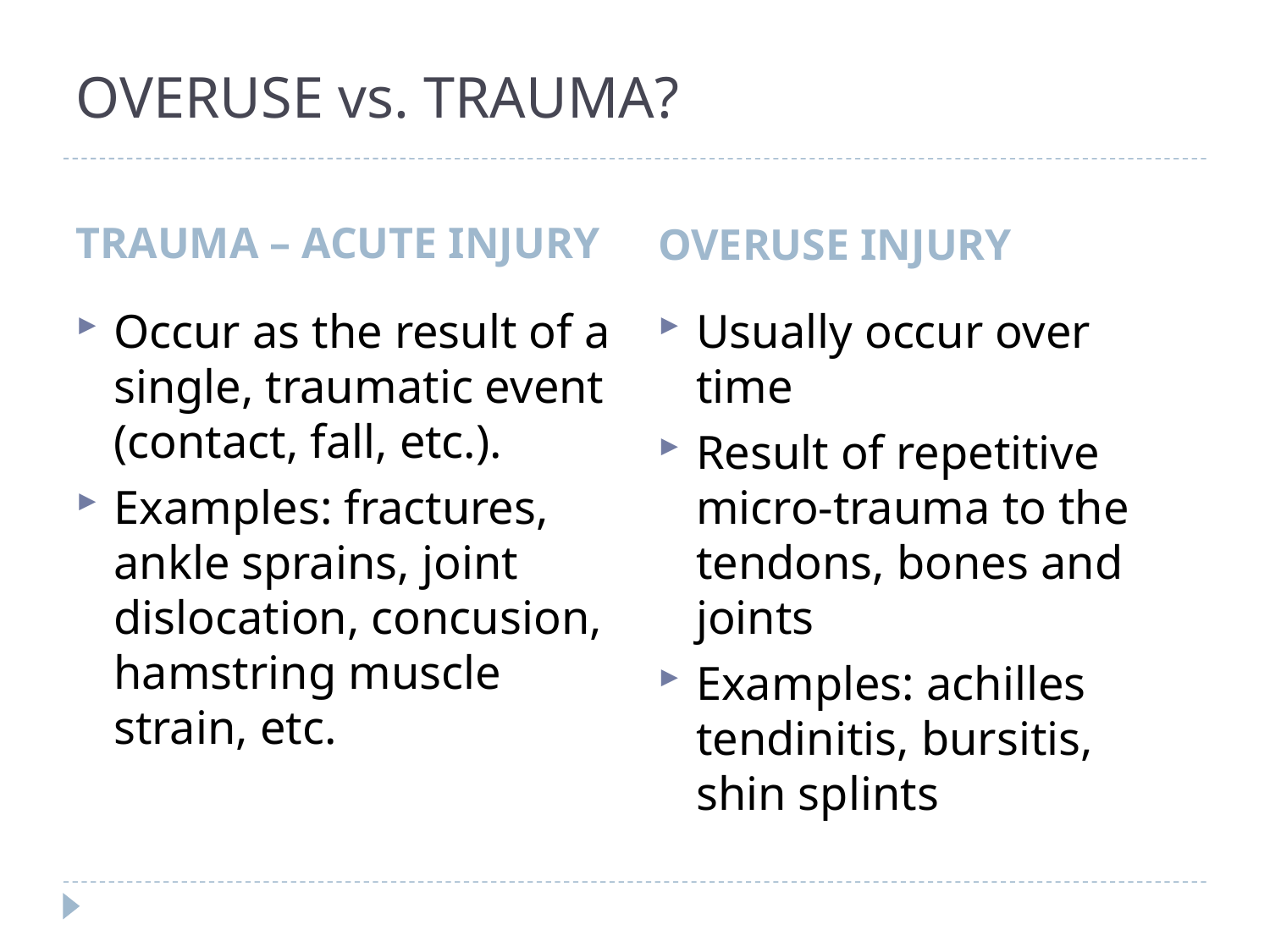

# OVERUSE vs. TRAUMA?
TRAUMA – ACUTE INJURY
OVERUSE INJURY
Occur as the result of a single, traumatic event (contact, fall, etc.).
Examples: fractures, ankle sprains, joint dislocation, concusion, hamstring muscle strain, etc.
Usually occur over time
Result of repetitive micro-trauma to the tendons, bones and joints
Examples: achilles tendinitis, bursitis, shin splints

## Slide 9
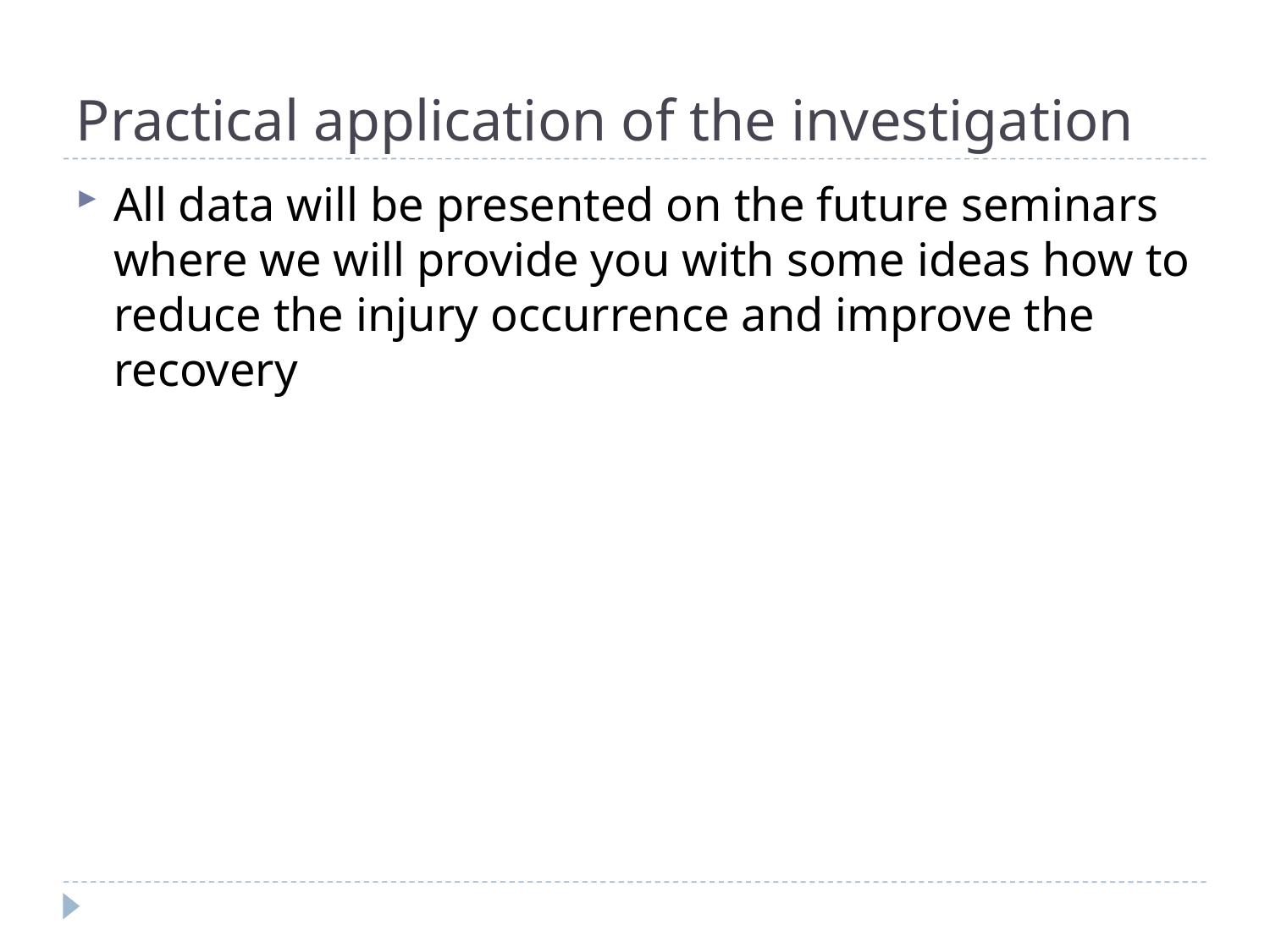

# Practical application of the investigation
All data will be presented on the future seminars where we will provide you with some ideas how to reduce the injury occurrence and improve the recovery

## Slide 10
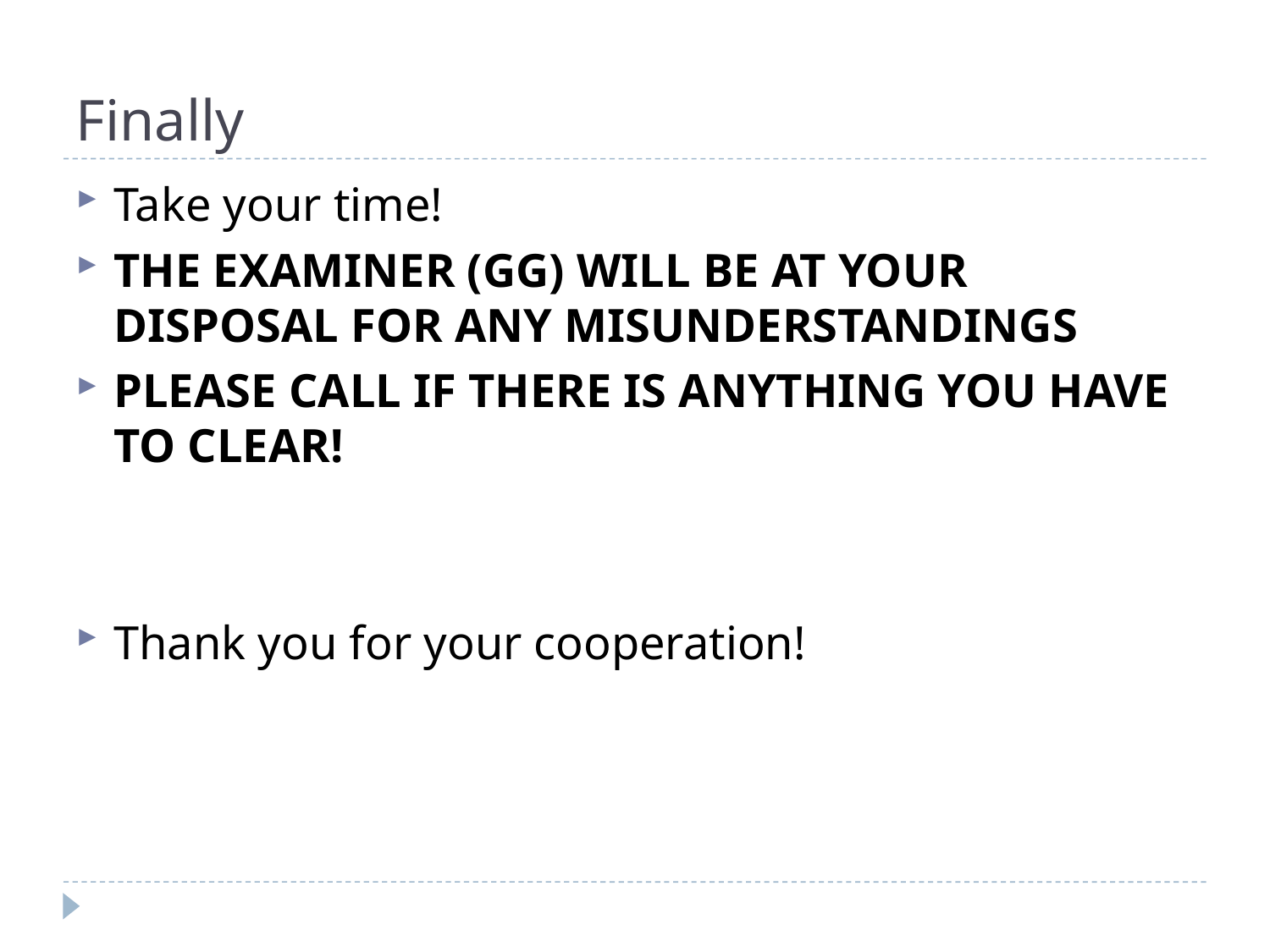

# Finally
Take your time!
THE EXAMINER (GG) WILL BE AT YOUR DISPOSAL FOR ANY MISUNDERSTANDINGS
PLEASE CALL IF THERE IS ANYTHING YOU HAVE TO CLEAR!
Thank you for your cooperation!
